# Benefits of an Immunogenic Personalized Neoantigen Nanovaccine in Patients with High‐Risk Gastric/Gastroesophageal Junction Cancer

**DOI:** 10.1002/advs.202203298

**Published:** 2022-11-09

**Authors:** Qin Liu, Yanhong Chu, Jie Shao, Hanqing Qian, Ju Yang, Huizi Sha, Lanqi Cen, Manman Tian, Qiuping Xu, Fangjun Chen, Yang Yang, Weifeng Wang, Kai Wang, Lixia Yu, Jia Wei, Baorui Liu

**Affiliations:** ^1^ The Comprehensive Cancer Centre of Drum Tower Hospital Medical School of Nanjing University and Clinical Cancer Institute of Nanjing University Nanjing 210008 China; ^2^ OrigiMed Shanghai 201114 China

**Keywords:** gastric/gastroesophageal junction cancer, nanovaccine, neoantigens, PD‐1 blockade, personalized vaccine, prevent postoperative cancer recurrence and metastasis

## Abstract

Personalized neoantigen vaccines have shown strong immunogenicity in clinical trial, but still face various challenges in facilitating an efficient antitumor immune response. Here, a personalized neoantigen nanovaccine (PNVAC) platform for adjuvant cancer immunotherapy is generated. PNVAC triggers superior protective efficacy against tumor recurrence and promotes longer survival than free neoantigens, especially when combined with anti‐PD‐1 treatment in a murine tumor model. A phase I clinical trial (ChiCTR1800017319) is initiated to evaluate the safety, immunogenicity, and prophylactic effect of PNVAC on preventing tumor recurrence in patients with high‐risk gastric/gastroesophageal junction cancer after adjuvant chemotherapy of postsurgical resection. The one‐ and two‐year disease‐free survival rates are significantly higher than historical record. PNVAC induces both CD4^+^ and CD8^+^ T cell responses as well as antigen‐experienced memory T cell phenotype. Furthermore, the immune response is persistent and remains evident one year after the vaccination. This work provides a safe and feasible strategy for developing neoantigen vaccines to delay gastric cancer recurrence after surgery.

## Graphical abstract

Personalized neoantigen vaccine can trigger specific anti‐tumor T cell responses. However, there are some concerns with the use of neoantigen vaccine, including unfavorable pharmacokinetic profile and poor antigen presentation. A universal nanovaccine platform is built to overcome the above challenges and it demonstrates superior protective efficacy against advanced gastric cancer recurrence in preclinical studies and a phase I clinical trial.

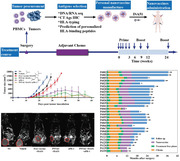

## 1 Introduction

Neoantigens encoded by tumor‐specific mutations are key targets of efficient T cell‐mediated immunity and antitumor immune responses.^[^ ^1^, ^2^ ^]^ Neoepitopes are highly immunogenic and recognized as excellent therapeutic candidates for cancer vaccines. Recent clinical trials for melanoma and glioblastoma revealed that peptide‐based neoantigen vaccines were safe and demonstrated promising signs of clinical efficacy and strong immunogenicity.^[^ ^3^, ^4^, ^5^ ^]^ However, peptide‐based neoantigen vaccines are generally challenged by tumor heterogeneity, early enzymatic degradation, a poor antigen‐presenting cells (APCs) and lymph nodes (LNs) trafficking.^[^ ^6^ ^]^ Nanotechnology‐based cancer vaccines have the potential to avoid rapid in vivo clearance of delivery immunomodulators to lymphoid organs and regulate the delivery of cell‐impermeable compounds to immune cells, thus improving the antigen uptake of APCs.^[^ ^7^, ^8^, ^9^ ^]^

Gastric carcinoma is the fifth most commonly diagnosed cancer and the third leading cause of cancer‐related death in the world.^[^ ^10^, ^11^ ^]^ The two‐year disease‐free survival (DFS) rate is ≈60% in patients with stage III gastric cancer treated by curative surgery followed by adjuvant chemotherapy.^[^ ^12^ ^]^ In the CLASSIC clinical trial study, the three‐year overall survival rate was only 33% for stage IIIB gastric cancer.^[^ ^13^ ^]^ Many patients with gastric cancer are diagnosed at an advanced stage with poor prognosis. There are no further recommendations in the National Comprehensive Cancer Network (NCCN) guidelines for gastric cancer patients undergoing D2‐radical gastroectomy followed by adjuvant chemotherapy.^[^ ^14^ ^]^ A novel approach is urgently needed to improve the generally poor prognosis of patients with high‐risk gastric/gastroesophageal junction (G/GEJ) cancer after adjuvant chemotherapy following surgical resection.

In this study, we generated a personalized neoantigen nanovaccine (PNVAC) without complicated chemical appendages. PNVAC elicited potent and durable antitumor immunity in a syngeneic murine forestomach cancer (MFC) model. In this first‐in‐human phase I clinical trial, we report the safety, prophylactic effect, and immunogenicity of PNVAC in patients with stage IIIB/IIIC/IVA G/GEJ cancer after adjuvant chemotherapy post‐surgery.

## 2 Results

### 2.1 Generation and Characterization of PNVAC

The neoantigens of the MFC cell line were identified by whole‐exome sequencing (WES). The binding affinity of mutant peptides to H_2_‐K^K^ was determined using netMHCpan v4.0. 9‐mer and 10‐mer mutant peptides with a binding affinity half‐maximum inhibitory concentration < 500 × 10^−9^ m or percentage rank < 2.0 were selected. Nine mutant epitopes for MFC cells were synthesized, including MFC‐1 (DEIVMFTLI), MFC‐2 (MELLGHGMV), MFC‐3 (VENVAWTHI), MFC‐4 (LEMNFYWSL), MFC‐5 (IEFIRKFAV), MFC‐6 (LEMNFYWSLV), MFC‐7 (MELTCSSTYV), MFC‐8 (YVENVAWTHI), and MFC‐9 (IDEIVMFTLI).

**Figure 1:**
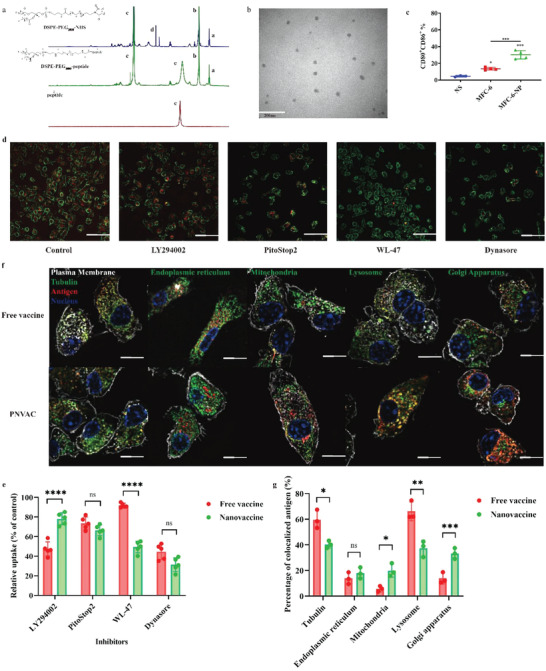
Personalized neoantigen nanovaccines (PNVAC) activate bone marrow‐derived dendritic cells (BMDCs) in vitro. a) ^1^H nuclear magnetic resonance (NMR) spectra of DSPE‐PEG_2000_‐NHS (vehicle), DSPE‐PEG_2000_‐peptide, and peptide (MFC‐7, MELTCSSTYV). The inset in DSPE‐PEG2000‐peptide shows the proton signal from methyl groups in antigen peptide, indicating portions of peptide were successfully conjugated into the copolymers. b) Transmission electron microscope image of PNVAC with a monolayer spherical morphology (20–30 nm diameter). Scale bar, 200 nm. c) Mouse BMDCs were incubated with normal saline (NS), free neoantigens (MFC‐6), and PNVAC (MFC‐6‐NP) for 48 h, and CD11c^+^CD80^+^CD86^+^ DCs were quantified using flow cytometry to assess DC maturation. *P*‐values were determined by one‐way ANOVA with Tukey's multiple comparisons test. *P = 0.018 (MFC‐6 vs NS), ***P < 0.001. d,e) NS (control) and four inhibitors were separately added into BMDCs (plasma membrane: red) before incubation with PNVAC (green). Fluorescence images were taken at 60 min after incubation by total internal reflection fluorescent microscope. Scale bar, 30 µm. Micropinocytosis inhibitor: LY294002, clathrin inhibitor: PitoStop2, caveolin inhibitor: WL‐47, dynamin inhibitor: dynasore. f,g) Fluorescence images were taken at 120 min after mouse BMDCs (plasma membrane: gray) incubation with free vaccine (red) or PNVAC (red) by total internal reflection fluorescent microscope. Tubulin, endoplasmic reticulum, mitochondria, lysosome, or Golgi apparatus: green. Scale bar, 10 µm.

Each neoantigen peptide was conjugated to amphiphilic lipids 1,2‐distearoyl‐sn‐glycero‐3‐phosphoethanolamine‐N‐[hydroxysuccinimidyl(polyethyleneglycol)] (DSPE‐PEG_2000_‐NHS) to synthesize nanoparticles and finally nine nanoparticles mixed together to generate PNVAC. ^1^H nuclear magnetic resonance and MALDI‐TOF mass spectrometry molecular weight characterization confirmed the successful conjunction of peptides (**Figure** **1**a; Figure S1a, Supporting Information). Dynamic light scattering analysis revealed that the particle size of PNVAC peaked at 20–30 nm and it was slightly negatively charged (Figure S1b,c, Supporting Information). The amphiphilic DSPE‐PEG_2000_‐peptides were self‐assembled with a core of DSPE and a shell of PEG_2000_‐peptides, as shown by transmission electron microscopy (Figure 1b). The size and polydispersity index of PNVAC remained stable for up to 96 h at either 4, 25, or 37 °C (Figure S1d, Supporting Information). Take MFC‐9 based nanoparticle as an example, it released more than 80% of the peptide after 96 h at 25 °C (Figure S1e, Supporting Information).

### 2.2 Activation of Dendritic Cells (DCs) by PNVAC

Among all nine neoantigens, five neoantigen‐based nanovaccines significantly induced the expression of the mature phenotypic markers CD80 and CD86 on CD11c^+^ DCs, compared to the free neoantigens‐treated group (Figure 1c; Figure S2a,b, Supporting Information). Taking the MFC‐6 neoantigen as an example, 34.3% of the CD11c^+^ DCs showed CD80^+^CD86^+^ in the PNVAC‐treated group, while only 15.1% of the CD11c^+^ DCs for the free MFC‐6 group did (Figure 1c; Figure S2b, Supporting Information).

Total internal reflection fluorescent microscope was used to demonstrate the uptake of PNVAC by bone marrow‐derived dendritic cells (BMDCs) in vitro. Fluorescence images taken every 20 min showed that dispersed free vaccine gradually aggregated and the fluorescence intensity in BMDCs gradually increased and presented dot distribution over time (Figure S3a, Supporting Information). On the contrary, PNVAC showed condensed point distribution at first and gradual dispersion (Figure S3b, Supporting Information). The fluorescence intensity of PNVAC in BMDCs dropped significantly after incubation with caveolin inhibitor WL‐47 and dynamin inhibitor dynasore, indicating uptake of PNVAC mainly depends on caveolin and dynamin (Figure 1d,e). Meanwhile, uptake of free vaccine is associated with micropinocytosis and dynamin (Figure S3c, Supporting Information; Figure 1e). Once ingested, free vaccine was mainly distributed in lysosomes and easily degraded (Figure 1f,g). However, part of PNVAC was shown to be located on the surface of Golgi apparatus, which is responsible for post‐translational modification and process of proteins, indicating nanotechnology is conducive to lysosomal escape and promote antigen presentation (Figure 1f,g).

### 2.3 Accumulation of PNVAC in LNs

**Figure 2:**
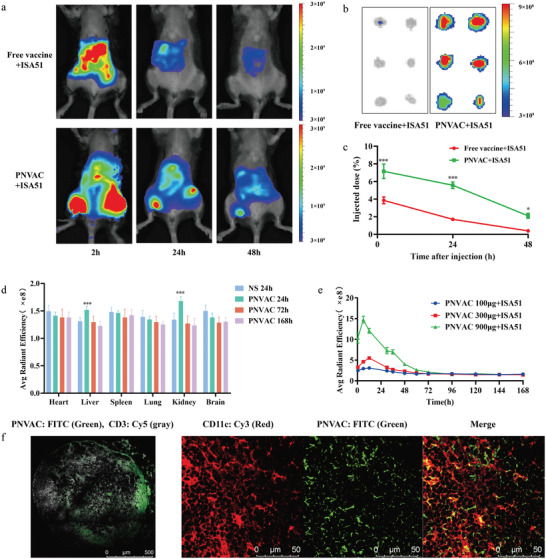
Personalized neoantigen nanovaccines (PNVAC) accumulate in lymph nodes (LNs). a–c) 615‐strain mice were injected subcutaneously with Cy5‐labeled neoantigen peptide MFC‐2 (free vaccine) or MFC‐2 based PNVAC mixed with Montanide ISA 51 (ISA 51) at the tail base, respectively (*n* = 3). a) Near‐infrared images of the biodistribution of control free vaccine or PNVAC in mice at 2, 24, and 48 h after subcutaneous injection. b) Near‐infrared images of inguinal LNs at 24 h post‐injection. c) Fluorescence semiquantification of peptide accumulation in LNs at different time points following subcutaneous injection of free vaccine or PNVAC. Quantitative IVIS data of the inguinal LNs were presented as percentage injected dose. *P*‐values were determined by two‐way ANOVA with Tukey's multiple comparisons test. ***P < 0.001, *P = 0.0438. d) NS or Cy5 labeled with PNVAC mixed with ISA 51 were administrated subcutaneously near the tail base of 615‐strain mice. Main organs (brain, heart, liver, spleen, lung, kidney) were collected and ground into a certain volume of single cell suspension at 24, 72, and 168 h after injection, respectively (*n* = 7). 100 µL of each sample were added into 96‐well plate to examine the fluorescence intensity and quantitative IVIS data were presented as average radiant efficiency. *P*‐values were determined by two‐way ANOVA with Tukey's multiple comparisons test. ***P < 0.001. e) 615‐strain mice were injected subcutaneously with 100, 300, or 900 µg Cy5‐labeled PNVAC mixed with ISA 51 near the tail base, respectively (*n* = 8). IVIS quantification of PNVAC concentration in blood were presented as average radiant efficiency. f) Localization of CD3^+^ T cells (gray) or CD11c^+^ DCs (red) and FITC‐labeled PNVAC (green) in LNs at 48 h after subcutaneous injection. Scale bar, 500 µm, 50 µm.

The Cy5‐labeled PNVAC or free neoantigens were subcutaneously injected near the tail base of 615‐strain mice to assess the biodistribution through fluorescence using an in vivo imaging system (IVIS). The fluorescence intensity decreased after injection in both groups, but PNVAC showed a stronger fluorescence signal than the free vaccines control group (**Figure** **2**a–c). The inguinal LNs were harvested at 24 h after injection and the fluorescence intensity of PNVAC tripled that of free vaccines (Figure 2b,c; *P* < 0.001). At the same time, we also detected brighter fluorescence signals in the liver (*P* < 0.001) and kidney (*P* < 0.001), but the fluorescence intensity of heart, lung, spleen, and brain remained undetectable (Figure 2d). After 72 h, there was no fluorescence signal detected in all organs. Due to the detection sensitivity of high‐performance liquid chromatography (HPLC), neoepitope can only be detected at high concentration (900 µg/mouse, Figure S1f, Supporting Information), but not at low and medium concentration (100 and 300 µg/mouse). Hence, the blood fluorescence intensity of mice was used to semiquantify the peripheral concentration of PNVAC. The half‐life of PNVAC in plasma was determined to be 24 h (Figure 2e). Compared with free vaccine, histological sections of the inguinal LNs indicated that PNVAC accumulated mainly in the subcapsular sinus and interfollicular areas (Figure 2f; Figure S3d, Supporting Information). Besides the altered biodistribution, we found that PNVAC spatially overlapped with DCs in the LNs (Figure 2f; Figure S3d, Supporting Information).

### 2.4 PNVAC Elicited Neoantigen‐Specific T Cells and Memory T Cells

**Figure 3:**
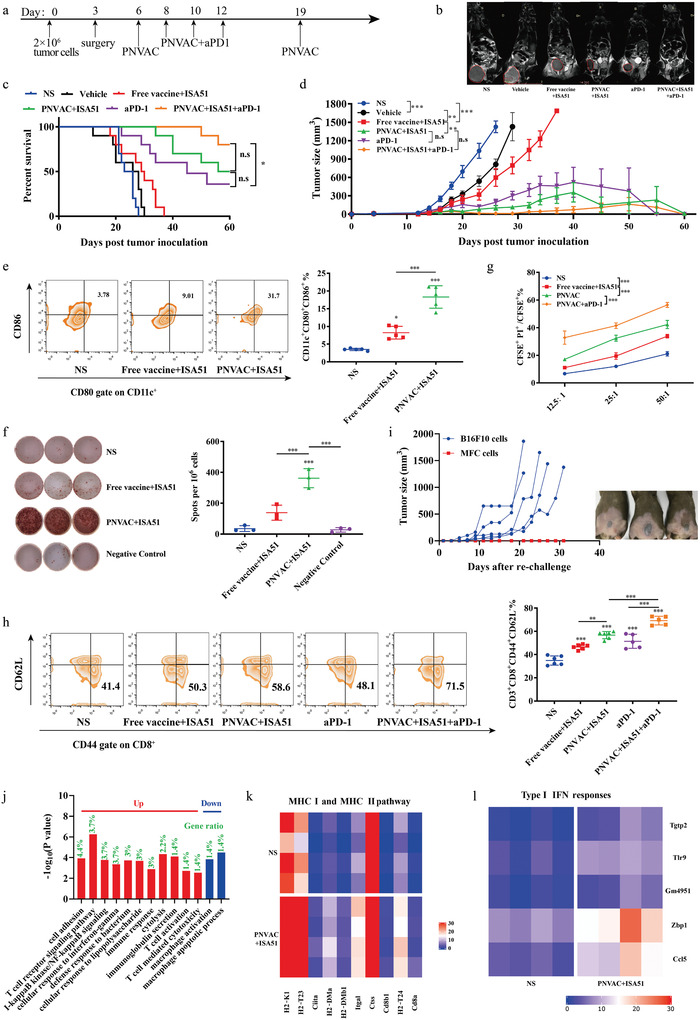
Personalized neoantigen nanovaccines (PNVAC) inhibit tumor growth and elicit specific T cell responses. a) Treatment schema. MFC cells (2 × 10^6^ cells per mouse) were subcutaneously injected into the abdomen of 615‐strain mice. The established tumors were removed when the tumor volume reached ≈100 mm^3^. Three days after surgery, the tumor‐bearing mice were randomly divided into six groups and injected subcutaneously with NS (normal saline), vehicle, free vaccine+ISA 51 (20 µg per peptide, 50 µL ISA 51 per mouse), PNVAC+ISA 51, aPD‐1 (anti‐PD‐1, 0.5 mg kg^−1^, intraperitoneally injected) or PNVAC+ISA 51+aPD‐1. Mice were immunized with PNVAC five times on the indicated days. b) MRI images of representative mice were acquired on day 25 and showed tumor regression after PNVAC vaccination. The tumor of each mouse is circled. c) Kaplan–Meier survival curves, and d) average tumor growth curves in different groups (*n* = 10). c) *P*‐values were determined by Log‐rank (Mantel–Cox) test. *P = 0.0263, n.s. not significant. d) *P*‐values were determined by two‐way ANOVA with Tukey's HSD multiple comparison post hoc test. ***P < 0.001, **P = 0.0014 (free vaccine+ISA 51 vs vehicle), **P = 0.0083 (PNVAC+ISA 51 vs Free vaccine+ISA 51), n.s. not significant. e–h) Mouse splenocytes were isolated one week after the last vaccination. e) Proportions of mature DCs (CD11c^+^CD80^+^CD86^+^) were analyzed using flow cytometry (*n* = 5). *P*‐values were determined by one‐way ANOVA with Tukey's multiple comparisons test. *P = 0.0104, ***P < 0.001. f) Splenocytes of each group were restimulated with RPMI 1640 medium (negative control) or free vaccine in vitro. IFN‐*γ* secretion was detected by ELISPOT (*n* = 3). *P*‐values were determined by one‐way ANOVA with Tukey's multiple comparisons test. ***P < 0.001. g) Splenocytes from mice in each group were isolated and incubated with CFSE‐labeled MFC cancer cells at effector‐to‐target ratios (E:T) of 12.5:1, 25:1, and 50:1. PI was added after 6 h incubation and the percentage of dead cells (CFSE^+^PI^+^) was analyzed using flow cytometry (*n* = 3). *P*‐values were determined by two‐way ANOVA with Tukey's HSD multiple comparison post hoc test. ***P < 0.001. h) Proportions of effector memory T cells (TEM, CD3^+^CD8^+^CD44^+^CD62L^−^) in mouse splenocytes analyzed using flow cytometry. *P*‐values were determined by one‐way ANOVA with Tukey's multiple comparisons test. ***P < 0.001, **P = 0.0013. i) MFC tumor‐bearing mice in PNVAC+ISA 51 group free of tumor recurrence or achieving complete regression after recurrence were subcutaneously rechallenged with B16F10 melanoma tumor cells (2 × 10^5^) in the left side of abdomen and MFC gastric cancer cells (2 × 10^6^) in the right side of abdomen 60 d post first inoculation. Shown are tumor growth curves for each mouse following the rechallenge (*n* = 5). Pictures of representative mice were taken on day 20 after rechallenge and tumors were circled. j) Pathways with significantly differentiated expression between NS and PNVAC+ISA 51 groups are presented based on the gene hits using RNA‐seq. Gene expression was clustered using log_10_(FKPM+1) and GO enrichment analysis. Differential gene expressions in k) MHC I and II pathway and l) type I IFN responses.

To validate the prophylactic and antitumor effects of PNVAC, a murine forestomach carcinoma model was established and immunized with vaccines after the growing tumor was resected with a positive margin (**Figure** **3**a; Figure S4a, Supporting Information). Magnetic resonance imaging (MRI) revealed that free neoantigens showed modest benefits against tumor recurrence (Figure 3b) compared to the blank control group, which was consistent with previous clinical studies.^[^ ^15^, ^16^, ^17^ ^]^ Montanide ISA 51 (ISA 51) is a water‐in‐oil emulsion, which has been widely used as a vaccine adjuvant in therapeutic and prophylactic vaccine trials.^[^ ^18^ ^]^ PNVAC+ISA 51 group substantially potentiated the protective efficacy against tumor recurrence and long survival time with approximately half of mice surviving at 60 d (Figure 3c,d; Figure S4b, Supporting Information), much better than the therapeutic responses achieved in free vaccine+ISA 51 group. Blood chemistry tests, body weights, and pathological sections of main organs revealed that PNVAC did not cause severe in vivo toxicity (Figure S5a–d, Supporting Information).

We then investigated whether the PNVAC elicited in vivo DC maturation and antigen‐specific T cell responses. After immunized with PNVAC, inguinal LNs of tumor‐bearing mice were harvested and digested into single‐cell suspensions for flow cytometry analysis. PNVAC was verified to promote APCs uptake and presentation of neoantigens and functionally induced more mature DCs in LNs (Figure S6a, Supporting Information). The splenic lymphocytes were collected one week after the last vaccination. The proportions of CD11c^+^CD80^+^CD86^+^ DCs were 9.01% and 31.7% in the free vaccine and PNVAC group, respectively (Figure 3e). Another DC mature marker CD40 was detected upregulation after PNVAC treatment as well (Figure S6b,c, Supporting Information). The proportion of CD11c^+^CD40^+^ tumor‐infiltrating DCs in PNVAC+ISA 51 group was 1.60‐fold higher than that in free vaccine‐treated group (Figure S6c, Supporting Information, free vaccine+ISA 51 vs PNVAC+ISA 51: 42.87 vs 68.67, *P* < 0.001). IFN‐*γ* enzyme‐linked immune absorbent spot (ELISPOT) showed that peptide‐specific IFN‐*γ* secretion by splenocytes increased significantly following PNVAC treatment (Figure 3f). Slightly larger proportion of spleen cytotoxic T cells (CD3^+^CD8^+^CD107a^+^, Figure S6d, Supporting Information) and that of tumor‐infiltrating cytotoxic T cells were both identified in PNVAC+ISA 51 group (Figure S6e, Supporting Information, free vaccine+ISA 51 vs PNVAC+ISA 51: 14.73 vs 20.47, *P* = 0.0177). The antitumor efficacy of the isolated splenocytes was tested using a carboxyfluorescein succinimidyl ester (CFSE)/propidium iodide (PI) labeling cytotoxicity assay. MFC cancer cells were stained with CFSE for 10 min at 37 °C in the dark. The splenocytes from the mice in each group were incubated with CFSE‐labeled MFC cancer cells at different effector‐to‐target ratios (E:T) to analyze the proportion of dead tumor cells (PI^+^CFSE^+^/CFSE^+^). The CD8^+^ T cells in PNVAC‐treated group were more effective at killing MFC cells than those of the normal saline (NS)‐treated and free vaccines‐treated groups (*P* < 0.001). The combination of PNVAC and anti‐PD‐1 potentiated killing tumor cell ability better than PNVAC alone (*P* < 0.001, Figure 3g).

Memory T cells are required for long‐lasting antitumor responses when direct killing is not sufficient to fully eradicate all tumor cells.^[^ ^19^, ^20^, ^21^ ^]^ PNVAC induced more effector memory CD8^+^ T cells (CD3^+^CD8^+^CD44^+^CD62L^−^) than the free peptides, particularly when combined with anti‐PD‐1 treatment (Figure 3h). The tumor‐bearing 615‐strain mice in PNVAC+ISA 51 group free of tumor recurrence or achieving complete regression after recurrence were simultaneously rechallenged with MFC cells in the right lower abdomen and the murine melanoma cells B16F10 in the left lower abdomen. The B16F10 cells rapidly grew into masses after implantation, while no MFC tumor growth was observed (Figure 3i), suggesting that PNVAC successfully induced immunological memory. To further investigate the altered tumor immune microenvironment, RNA sequencing (RNA‐seq) analysis was performed on tumor tissues from the PNVAC+ISA 51 and control NS group. In total, 122 genes were upregulated while 13 genes were downregulated. Cluster analysis revealed that the differentially expressed genes were found to be mainly associated with adaptive immunity (Figure 3j). Ten genes correlated with activated MHC class I and II antigen presentation pathways and five genes correlated with the type I IFN response upregulated were significantly upregulated (Figure 3k,l). However, more MDSCs accumulated in the tumor microenvironment after PNVAC treatment (Figure S6f, Supporting Information). The expression of PD‐1 on neoantigen‐specific CD3^+^ T cells following PNVAC stimulation suggested the exhaustion of these T cells (Figure S6g, Supporting Information). PNVAC combined with anti‐PD‐1 treatment thus potentiated the immunotherapeutic efficacy of PNVAC or anti‐PD‐1 alone (Figure 3c,d).

### 2.5 Clinical Outcomes and Immune Responses of PNVAC‐Treated G/GEJ Cancer Patients

**Figure 4:**
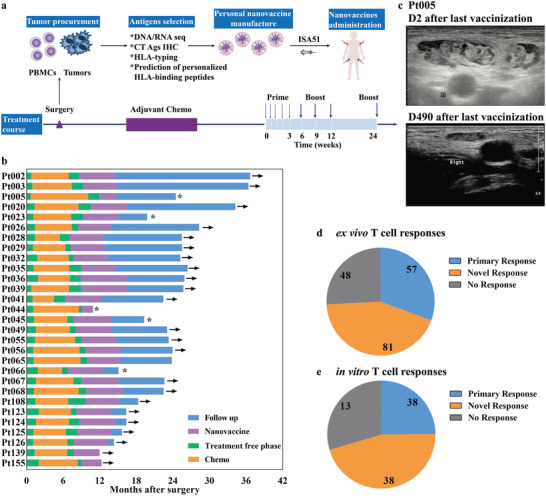
Generation of an individualized, multiepitope neoantigen nanovaccine (PNVAC) in patients with G/GEJ cancer. a) Tumor somatic mutations of gastric cancer were identified using WES and genes expression was confirmed using RNA‐seq. The expression of cancer‐testis antigens (CTAs) was tested using immunochemical staining. Immunized neoantigens were identified on the basis of HLA binding affinity predictions and manufactured into PNVAC. The prepared PNVAC and adjuvant Montanide ISA 51 were injected subcutaneously at four injection sites. b) Clinical outcomes of 29 vaccinated G/GEJ cancer patients after surgery until the disease was progressed or the cut‐off date, December 31, 2020. Asterisk represents patients with progressed diseases and arrow represents disease free patients. c) Ultrasound images of lymph nodes after PNVAC injection. Summary of T cells immune responses to PNVAC: d) ex vivo and e) in vitro stimulation. The patients’ immune responses to PNVAC were evaluated by detecting and monitoring the IFN‐*γ* released from peripheral blood mononuclear cells (PBMCs) using CBA assay. The IFN‐*γ* expression of mutant peptide‐stimulated PBMCs greater than twice the negative control were considered to have positive immune responses.

Given the biological and functional significance of PNVAC, a phase I clinical trial was initiated to study the safety, immunogenicity, and prophylactic effect on tumor recurrence of PNVAC in patients with stage IIIB/IIIC/IVA gastric cancer after surgery. The neoantigens were selected based on tumor‐specific mutations identified using WES and RNA‐seq. The cancer‐testis antigens (CTAs) were selected according to the immunohistochemical staining of the resected tumor tissues and HLA‐binding affinity prediction. PNVAC combined with adjuvant ISA 51 was administered subcutaneously to the corresponding 29 patients following completion of adjuvant chemotherapy (capecitabine/S‐1+oxaliplatin or S‐1+docetaxel) on days 1, 4, 8, 15, 22, 43, 64, 85, and 169 (**Figure** **4**a). Four injections for four patients were delayed due to COVID‐19‐related restrictions.

Of the 30 enrolled patients, one patient withdrew due to disease progression during adjuvant chemotherapy. The patients’ median age was 57 years old (range: 34–75 years), and their Eastern Cooperative Oncology Group performance status was either 0 or 1 (Table S1, Supporting Information). We detected an average of 186 somatic variants with a median of 153 single‐nucleotide variants per tumor using WES, and RNA‐seq was performed to confirm the gene expressions. A median of 183.3 HLA binders with an average half‐maximum inhibitory concentration < 500 × 10^−9^ m were predicted per tumor. The median of detected somatic mutations was 153 (range: 75–158). Each patient received ≈9 personalized immunizing peptides (range: 6–13) with lengths of 9–15 amino acids (Table S2, Supporting Information). The median time from the last adjuvant chemotherapy cycle to vaccine administration was 5.1 weeks. In total, 29 patients received an initial vaccination and 27 patients completed the full series, comprising five priming shots and four booster shots. The repeated doses were well tolerated and no severe adverse events occurred. Twelve patients (41.4%) developed grade 1 local skin reactions with redness, swelling, or subcutaneous indurations at the injection sites and two patients (6.9%) developed grade 2 local skin reactions. Five patients (17.2%) had a grade 1 or 2 fever after injection. Two patients (6.9%) developed grade 2 myalgia (Table S3, Supporting Information). The median follow‐up time after surgery was 26.4 months (range: 15–39.7 months). The one‐ and two‐year survival rates were 96.6% (28/29) and 82.4% (14/17), respectively (Figure 4b). Only one patient demonstrated an anastomotic recurrence at the beginning of vaccination within one year after surgery. The clinical outcome was superior to the previous study, which reported a disease‐free survival of 17 months for stage IIIB and 16 months for stage IIIC gastric cancer patients treated with a D2‐radical gastroectomy followed adjuvant chemotherapy.^[^ ^22^ ^]^ The LNs of patient 005 were observed to be swollen and draining on day 2 after the last shot, but they had returned to normal on day 490 (Figure 4c), indirectly indicating the efficient LNs uptake of the administered PNVAC.

**Figure 5:**
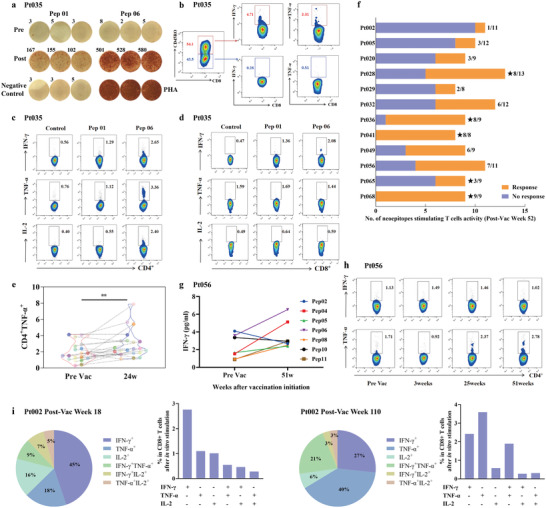
Personalized neoantigen nanovaccines (PNVAC) induce multifunctional circulating CD4^+^ and CD8^+^ T cell responses in gastric cancer patients. a) IFN‐*γ* ELISPOT of patient 035 PBMCs stimulated with individualized peptides to assess the neoantigen‐specific T cell responses. No peptide media and phytohemagglutinin (PHA) were used as negative and positive controls, respectively. b) ICS of PBMCs after neoantigens stimulation. PBMCs were pre‐gated based on CD3^+^ and CD8^+^ T cells. Cytokine‐producing neoantigen‐specific T cells expressed CD45RO, demonstrating an antigen‐experienced memory T cell phenotype. c) In vitro measurement of intracellular IFN‐*γ* ^+^, TNF‐*α* ^+^, and IL‐2^+^ CD4^+^ T cells in PBMCs from patient 035 who was treated with individualized peptides. d) In vitro measurement of intracellular IFN‐*γ* ^+^, TNF‐*α* ^+^, and IL‐2^+^ CD8^+^ T cells in patient 035 PBMCs which were stimulated with individualized peptides. e) Median percentage of cytokine production across 21 patients by CD4^+^ T cells stimulated in vitro with immunized peptides. Data were analyzed by paired two‐tailed Student's *t*‐test. **P = 0.0048. f) Nanovaccines induced persistent immune responses, as indicated by the level of IFN‐*γ* in PBMCs at week 52 after nanovaccines therapy among 12 patients. The number of responsive peptides for each patient is represented as orange stacked column at week 52 and the blue stacked column represents the number of no‐responsive peptides. g) The PBMCs of patient 056 at different time points were restimulated with immunized peptides overnight. Then the IFN‐*γ* concentration in culture supernatant was measured by IFN‐*γ* CBA assay. h) ICS of IFN‐*γ* and TNF‐*α* cytokine‐producing CD4^+^ T cells in PBMCs from patient 056 after one round prestimulation at the specific time point. i) Pie charts show that total CD8^+^ T cell responses positive for 1 or 2 cytokines (IFN‐*γ*, TNF‐*α*, or IL‐2) at week 18 and week 110 after initial vaccination in patient 002. Bar graphs show the absolute frequencies of ASP pool‐reactive CD8^+^ T cells producing one or two cytokines.

The patients’ immune responses to PNVAC were evaluated by detecting and monitoring the IFN‐*γ* released from peripheral blood mononuclear cells (PBMCs) using various assays, including ex vivo or in vitro cytometric bead arrays (CBA), IFN‐*γ* ELISPOT assay, and intracellular cytokine staining (ICS). The immunized antigens elicited T cell responses in all patients and 93.1% of patients generated novel immune responses. Furthermore, 77.8% (214/275) of immunized peptides induced positive T cells because the IFN‐*γ* secretion was double that of the medium (no peptide) control (Figure 4d,e; Figures S7–S9, Supporting Information). When monitored by an ex vivo IFN‐*γ* CBA assay, ≈30.6% (57/186) of the immunized neoantigens induced primary responses and 43.5% (81/186) induced novel antigen reactive responses after repeated PNVAC treatment (Figure 4d; Figures S7 and S8, Supporting Information). In some cases, the reactivity of T cells was assessed using in vitro IFN‐*γ* CBA assay. The primary and novel responses to the given neoantigens were found to be 42.7% (38/89) and 42.7% (38/89), respectively, when the immune response was evaluated by in vitro stimulation (Figure 4e; Figure S9, Supporting Information). Immunizing peptides 01 and 06 (Pep01 and Pep06, respectively) were used to test the immune response of patient 035 after vaccination. In vitro IFN‐*γ* ELISPOT showed that IFN‐*γ* secretion by PBMCs against Pep01 and Pep06 were undetectable before the vaccination but evident at six weeks following treatment by PNVAC (**Figure** **5**a, Pep01: 3.7 vs 141.3, *P* = 0.0022; Pep06: 5.0 vs 536.3, *P* < 0.001). When the immunizing peptides were mixed into a peptide pool to stimulate PBMCs, memory CD8^+^ T cells were the dominant subpopulation of the IFN‐*γ*‐producing antigen‐reactive CD8^+^ T cells (Figure 5b). In vitro ICS showed CD4^+^IFN‐*γ* ^+^ T cells were elicited to about 2.30‐folds by Pep01 and 4.73‐folds by Pep06 and CD8^+^IFN‐*γ* ^+^ T cells were initiated to about 2.89‐folds by Pep01 and 4.43‐folds by Pep06 (Figure 5c,d). The proportion of TNF‐*α* producing CD4^+^ T cells was significantly elevated in 21 patients at 24 weeks after vaccination (Figure 5e). CD4^+^IFN‐*γ* ^+^, CD8^+^IFN‐*γ* ^+^, and CD8^+^TNF‐*α* ^+^ T cell frequencies tended to increase after vaccination, although the differences were not statistically significant (Figure S10a–c, Supporting Information). Among 12 patients, 53.3% of the immunized neoepitopes retained detectable T cell activity one year after vaccination (Figure 5f). The PBMCs of patient 056, showed stronger reactivity against five out of seven immunizing peptides at ≈51 weeks after PNVAC treatment (Figure 5g). 66.7% (6/9) immunized mutant antigens elicited significant T‐cell specific antigen response for patient 036 at 63 weeks after PNVAC treatment (Figure S10d, Supporting Information). ICS also demonstrated that the development of a persistent immune response following PNVAC vaccination (Figure 5h). Taking patient 002 as an example, more than 20% of the neoantigen‐reactive CD8^+^ and CD4^+^ T cells were polyfunctional at 18 weeks post vaccination, as detected by the secretion of one or two inflammatory cytokines (Figure 5i; Figure S11, Supporting Information). In addition to exhibiting effectiveness, the immune responses induced by PNVAC were persistent, as indicated by the increasement of polyfunctional CD8^+^ and CD4^+^ T cells observed at 110‐week post vaccination (Figure 5j; Figure S11, Supporting Information).

**Figure 6:**
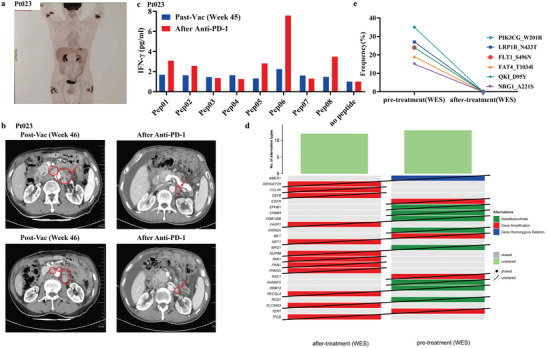
Clinical and immune responses to anti‐PD‐1 blockade alone in patient 023 who relapsed after treatment with personalized neoantigen nanovaccines (PNVAC). a) In patient 023, PET/CT scan revealed inflammatory uptake at the vaccinated sites. b) Computed tomography (CT) scans showed that after three doses of anti‐PD‐1 blockade immunotherapy, multiple metastatic lymph nodes shrank, but ascites appeared. c) The neoantigen‐specific T cell responses were enhanced after PD‐1 blockade in patient 023 who relapsed after vaccination. d) Comparison of somatic mutations between primary tumor and relapsed lesion detected using WES. e) Comparison of gene mutations corresponding to immunized neoantigens between primary tumor and progressed lesion in patient 023.

Besides analyzing the above patients that were disease‐free after PNVAC treatments, one patient with continued disease progression was also studied. Patient 023, a 67‐year‐old man with PD‐L1 negative stage IIIC gastric carcinoma, received PNVAC after six cycles of adjuvant chemotherapy. Four weeks after the first vaccination, he developed grade 2 local skin reactions with subcutaneous indurations. A positron emission tomography/computed tomography (PET/CT) scan revealed concurrently increased FDG uptake in subcutaneous indurations and residual stomach (**Figure** **6**a), indicating the active inflammation of injection sites. Approximately 46 weeks after vaccination, the disease progressed with retroperitoneal lymphadenopathy (Figure 6b). After anti‐PD‐1 antibody monotherapy, inconsistent responses were observed, with apparent shrinking of the swollen LNs and new‐onset ascites as well as anastomosis relapse (Figure 6b). Immune responses to each immunizing peptide were observed at the beginning of relapse (week 46) and were noticeably enhanced in five out of the eight immunizing peptides after treatment with an anti‐PD‐1 antibody (Figure 6c). A previous study of personalized neoantigen vaccination reported that two out of six melanoma patients who developed recurrent disease in the trial experienced expansion of neoantigen‐specific T cells and achieved complete regression after anti‐PD‐1 treatment.^[^ ^5^ ^]^ An enhanced immune response was also detected in patient 023 following PD‐1 blockade, suggesting the synergistic effects of neoantigen vaccine and anti‐PD‐1 therapy, which is consistent with the preclinical findings. Re‐biopsy was performed under endoscopy after the development of anastomotic obstruction. Interestingly, the progressed lesion sample after anti‐PD‐1 therapy was detected to have entirely different gene mutations compared to the previous surgical sample (Figure 6d) and none of the mutations corresponding to the patient's immunized neoantigens were detected in the progressed lesion (Figure 6e).

## 3 Discussion and Conclusion

Conventional tumor therapeutic vaccines usually select tumor‐associated proteins that are highly expressed by tumor cells as targets, but showed limited therapeutic efficacy because the proteins are also expressed by nonmalignant cells.^[^ ^23^, ^24^, ^25^ ^]^ Neoepitopes derived from somatic mutation can be processed and presented on MHC molecules and have a high affinity to T cell receptors.^[^ ^26^, ^27^ ^]^ CTAs, which are highly expressed in the tumors and testis but not expressed in normal tissues, are also favorable cancer vaccine targets.^[^ ^28^, ^29^ ^]^ Insufficient trafficking to lymphoid organs as well as delivery antigens to immune cells are responsible to limited clinical efficacy.^[^ ^30^, ^31^ ^]^ The number of tumor‐infiltrating T cells in the PNVAC‐treated group was significantly higher than that of the free vaccine‐treated group, suggesting that the neoantigen‐specific T cells generated by PNVAC are capable of trafficking to tumor sites. The IFN‐*γ*‐producing neoantigen reactive T cells highly expressed memory T‐cell phenotypic markers, indicating that patients with prior PNVAC treatment developed durable long‐term antitumor control. As a result, the administration of nanovaccines triggered a more significant tumor shrinking, and a longer survival time than traditional peptide‐based vaccines. Adjuvants are conventionally used to improve immunogenicity clinically.^[^ ^32^ ^]^ However, the discrepancy between antigen peptides and adjuvants with regard to their pharmacokinetics and physicochemical properties is a major obstacle in the development of therapeutic cancer vaccines.^[^ ^33^, ^34^ ^]^ For a vaccine adjuvant, we used topically applied ISA 51. By strategically loading multiple neoantigens and adjuvants in the same nanoparticle, the PNVAC mediated efficient antigen and adjuvant codelivery to LNs and APCs. In the PNVAC‐treated group, we found that genes related to antigen processing and type I IFN signaling were upregulated, suggesting that PNVAC promoted antigen presentation and process and potent T cell cytotoxicity to targeted tumor cells. The mice whose tumors were eradicated by PNVAC were still tumor‐free after rechallenge because PNVAC augmented neoantigen‐specific and effector memory T cell responses.

Resected stage IIIB–IVA gastric cancer is highly incurable, with high risks of recurrence and metastasis. In addition to the current strategies of definitive surgery and adjuvant chemotherapy, a novel strategy is needed to improve the poor prognosis of gastric cancer. For the first time, we integrated the personalized neoantigens/CTAs with this novel vaccine platform to generate PNVAC. The PNVAC platform not only showed an improved LN‐targeting capacity and a significant CD4^+^ and CD8^+^ T cells mediating immune response in the preclinical studies, but also clearly prolonged DFS in patients with high‐risk G/GEJ cancer. T cell responses against 77.8% immunized neoepitopes were detected in the enrolled 29 patients, indicating certain epitope prediction accuracy in a large cohort of patients and the possibility that a certain frequency epitope‐specific T cells were present before vaccination. Epitope spreading in the previous study was associated with prolonged progression‐free survival.^[^ ^35^ ^]^ In our study, most patients developed obvious epitope spreading because neoantigen‐directed responses were enhanced after PNVAC treatment.

Overall, we demonstrated that a personalized neoantigen‐based nanovaccine is safe and feasible for combatting gastric carcinomas, which typically are relatively unresponsive to PD‐1 inhibitors.^[^ ^36^, ^37^, ^38^ ^]^ A larger randomized clinical trial is needed to confirm and validate the possibility of neoantigen nanovaccine design for G/GEJ cancer patients in future.

## 4 Experimental Section

### Study Design

This study was designed to generation a personalized neoantigen nanovaccine platform as a monotherapy and as a combination therapy with PD‐1 blockade for adjuvant cancer immunotherapy. The antitumor efficacy and immune response of PNVAC in murine gastric cancer model and in a phase I clinical trial in high‐risk gastric/gastroesophageal junction cancer after adjuvant chemotherapy postsurgical resection were evaluated. Sample sizes were determined on the basis of the authors’ previous experience and previous study. Mice were randomized into various groups before treatment on the basis of tumor size and body weight. The investigators were not in a blinded fashion. In preclinical study, all experiments were run at least in triplicate. All experiments were conducted in accordance with the relevant guidelines and regulations for animal research at the Affiliated Nanjing Drum Tower Hospital of Nanjing University Medical School (2018AE01006).

### Neoantigen Prediction

Tumor tissue specimens and blood samples were collected from MFC tumor‐bearing mice and WES was performed (Shanghai Biotecan Pharmaceuticals Co., Ltd., Shanghai, China). The nonsynonymous mutations identified were sorted according to frequency and the PubMed nucleotide tool was used to identify amino acids of wild‐type proteins. Nine mutant epitopes were selected as target peptides for the vaccine design according to the predicted major histocompatibility complex (MHC)‐I H‐2K^k^ binding affinity.

### Generation and Characterization of PNVAC Nanovaccines

DSPE‐PEG_2000_‐NHS and individual peptides at a molar ratio of 1:1.5 were mixed in PBS. The amphiphilic polymers were purified through dialysis, lyophilized, and stored at −80 °C for further use. The characterizations including size, zeta potential, stability, and morphology were assessed.

### Biodistribution Study

The distribution of PNVAC was evaluated by in vivo fluorescence imaging using an optical and X‐ray small animal imaging system (IVIS Lumina, Perkin Elmer, Germany). Cy5‐labeled free vaccines and PNVAC were adjusted to equal Cy5 concentration and mixed with adjuvant ISA 51 and subcutaneously injected into separate 615‐strain mice (20 µg per peptide). The fluorescence signals were examined at 2, 24, and 48 h. The inguinal LNs were collected to assess the draining LNs targeting. For the pharmacokinetic study, the doses of neoantigens were 100, 300, or 900 µg. Blood and main organs (brain, heart, liver, spleen, lung, kidneys) were collected and analyzed.

### Tumor Model—Protective Immunity

To establish a gastric cancer model, 2 × 10^6^ MFC cells were injected subcutaneously into mice. Tumors were allowed to grow to a volume of ≈100 mm^3^. Subcutaneous dissection was kept to a minimum to avoid a large pocket of potential space. Tumors were transected sharply, leaving a 0.1 mm thick remnant. The mice were randomly divided into six groups and injected subcutaneously with NS, blank vehicle, free vaccines, nanovaccines, anti‐PD‐1 antibody (5 mg kg^−1^, injected intraperitoneally), or nanovaccines + anti‐PD‐1 (injected intraperitoneally). The size of the tumors were measured in an unblinded manner with calipers every 2 d and their volume was calculated using the equation (*a* ^2^ × *b*)/2 (*a*, width; *b*, length). The mice were euthanized upon exhibiting signs of impaired health or when the tumor length exceeded 15 mm.

### Tumor Model—Rechallenge Model

Tumor‐bearing mice cured by PNVAC were rechallenged with MFC tumor cells and B16F10 melanoma cells on opposite sides of the abdomen.

### Clinical Trial Design

Between 2018 and 2021, a single‐arm, open‐label first‐in‐human trial (ChiCTR1800017319) was conducted at the Cancer Centre of Nanjing Drum Tower Hospital in China and in accordance with the Declaration of Helsinki, International Conference on Harmonization/GCP guidelines. Patients were enrolled from July 2018 to July 2020. The enrollment dates for the first and last patient were July 26, 2018 and July 02, 2020, respectively. Data analysis was performed with data cutoff of December 31, 2020. Patients with an Eastern Cooperative Oncology Group score of 0–1 and a life expectancy of at least six months were enrolled. Patient identification logs were kept strictly confidential by the investigators (see the Chinese Clinical Trial Registry, http://www.chictr.org.cn/showproj.aspx?proj=27889, for details on the eligibility criteria). All patients provided written informed consent before enrollment. Patient‐specific neoantigens were selected on the basis of tumor‐specific mutations identified by WES and RNA‐seq (a process described in Materials and Methods of the Supporting Information). CTAs were tested using immunohistochemical staining and HLA‐binding affinity prediction. The customized peptides encoding individual neoantigens were prepared in a pharmacy under GMP conditions.

Patients with Stage IIIB/IIIC/IVA G/GEJ cancers who completed or could not tolerate six cycles of adjuvant chemotherapy (capecitabine/S‐1/oxaliplatin or S‐1/docetaxel) were treated with PNVAC. PNVAC was designed and manufactured to be administered during adjuvant chemotherapy. PNVAC treatment started within two months after the last adjuvant chemotherapy cycle. Each patient received a 500 µg dose per peptide of PNVAC by subcutaneous injection on days 1, 4, 8, 15, 22, 43, 64, 85, and 169, along with adjuvant chemotherapy in the form of ISA 51 and 100 µg GM‐CSF. Cyclophosphamide (200 mg m^−2^) was administered before PNVAC injection on days 1, 22, 43, 64, 85, and 169. PBMCs were isolated using a Ficoll density gradient centrifugation of heparinized blood samples from the patients and stored at −80 °C for immune response measurements on days 1, 22, 43, 64, and 169 before each dose of the vaccines was administered.

### Ex Vivo and In Vitro Evaluation of the Neoantigens’ Immunogenicity

Briefly, frozen PBMCs were thawed and cultured in culture AIM‐V medium supplement with 10% FCS. For ex vivo analysis with the IFN‐*γ* Flex Set, 2 × 10^5^ PBMCs were plated in 96‐well cell culture plates with individual peptides (25 µg mL^−1^) and incubated overnight; the supernatants were then harvested for further testing. The no‐peptide media and stimulus phytohemagglutinin were used as negative and positive controls, respectively. For in vitro stimulation of antigen‐specific T cells, 25 ng mL^−1^ IL‐7 was added to the culture medium and 20 U mL^−1^ IL‐2 was added on day 3. Half of the medium with additional cytokines was replaced every 3 d. After 1 d of stimulation, cells were harvested for further immune response analysis. The IFN‐*γ* expression of mutant peptide‐stimulated PBMCs greater than twice the negative control were considered to have positive PBMC reactivity.

### Statistical Analyses

For all experiments, biological replicates were performed unless otherwise stated. Comparisons between two groups were analyzed using paired two‐tailed Student's *t*‐test. One‐way analysis of variance (ANOVA) or two‐way ANOVA was performed to compare several groups. Survival benefit was determined with the log‐rank test. *P ≤ 0.05, **P ≤ 0.01, and ***P ≤ 0.001 were considered statistically significant.

## Conflict of Interest

The authors declare no conflict of interest.

## Supporting information

Supporting Information

Supplemental Table 1

Supplemental Table 2

Supplemental Table 3

## Data Availability

The data that support the findings of this study are available in the supplementary material of this article.
