# Supplementary material for: Benefits of an Immunogenic Personalized Neoantigen Nanovaccine in Patients with High‐Risk Gastric/Gastroesophageal Junction Cancer
Source: Adv Sci (Weinh). 2022 Nov 9;10(1):2203298. doi: 10.1002/advs.202203298 (PMC9811442; doi:10.1002/advs.202203298)
Supplement: Supplementary file 1 — Supporting Information [file ADVS-10-2203298-s002.pdf]

ADVANCED SCIENCE
Open Access

Supporting Information

for *Adv. Sci.*, DOI 10.1002/advs.202203298

Benefits of an Immunogenic Personalized Neoantigen Nanovaccine in Patients with High-Risk Gastric/Gastroesophageal Junction Cancer

Qin Liu, Yanhong Chu, Jie Shao, Hanqing Qian, Ju Yang, Huizi Sha, Lanqi Cen, Manman Tian, Qiuping Xu, Fangjun Chen, Yang Yang, Weifeng Wang, Kai Wang, Lixia Yu, Jia Wei and Baorui Liu**

Supporting Information

Title: Benefits of an immunogenic personalized neoantigen nanovaccine in patients with high-risk gastric/gastroesophageal junction cancer

Qin Liu[#], Yanhong Chu[#], Jie Shao, Hanqing Qian, Ju Yang, Huizi Sha, Lanqi Cen, Manman Tian, Qiuping Xu, Fanjun Chen, Yang Yang, Weifeng Wang, Kai Wang, Lixia Yu, Jia Wei, Baorui Liu**

This file includes:

Materials and Methods

Figure S1. Characterization of Personalized neoantigen nanovaccines (PNVAC).

Figure S2. Personalized neoantigen nanovaccines (PNVAC) activate bone marrow- derived dendritic cells (BMDCs).

Figure S3. In vitro uptake of personalized neoantigen nanovaccines (PNVAC) by bone marrow-derived dendritic cells (BMDCs).

Figure S4. Personalized neoantigen nanovaccines (PNVAC) inhibit tumor growth.

Figure S5. Safety of personalized neoantigen nanovaccines (PNVAC).

Figure S6. Personalized neoantigen nanovaccines (PNVAC) induce DCs maturation and improve tumor microenvironment.

Figure S7. Demonstration of epitope spreading.

Figure S8. Demonstration of epitope spreading.

Figure S9. Demonstration of epitope spreading.

Figure S10. The neoantigen specific T cell responses.

Figure S11. CD4+ T cell responses in patient 002.

Figure S12. Gating strategy.

Other Supplementary Materials for this manuscript includes the following:

Table S1. Clinical characteristics of patients.

Table S2. Individualized neoantigen for each patient.

Table S3. Summary of adverse events.

The supplement file. The clinical trial protocol.

Materials and Methods

Tumor cell line and mice: MFC murine forestomach carcinoma cell line (Shanghai Institute for Biological Science, China) was cultured in RPMI 1640 medium with 10% fetal bovine serum (FBS) at 37℃under an atmosphere of 5% CO2. Cells were sub-cultured at 80% confluence approximately every 2 to 3 days at a split ratio of 1:3. Female 615-strain mice (6-8 weeks old) were purchased from Shanghai Super-B&K Laboratory Animal Corp. Ltd. (Shanghai, China).

Materials : The antibodies in this manuscript include Mouse CD3 FITC (clone 500A2) Biolegend, 152304; Mouse CD4 PE (clone GK1.5) Biolegend, 100408; Mouse CD4 PE/Cy7 (clone GK1.5) Biolegend, 100422; Mouse CD8 PE/Cy5 (clone 53-6.7) Biolegend, 100710; Mouse CD8 APC (clone 53-6.7) Biolegend, 126614; Mouse CD11c FITC (clone N418) Biolegend, 117306; Mouse CD80 APC (clone 16-10A1) Biolegend, 104714; Mouse CD86 PE (clone A17199A) Biolegend, 159204; Mouse CD40 PE (clone FGK45) Biolegend, 157505; Mouse CD107a PE (clone 1D4B) Biolegend, 121611; Mouse CD44 PE (clone IM7) Biolegend, 103008; Mouse CD62L APC (clone MEL-14) Biolegend, 104412; Mouse PD-1 APC (clone 29F.1A12) Biolegend,135240; Mouse tubulin antibody (α-tubulin) Abcam, ab7291; Mouse Endoplasmic reticulum antibody (PDI) CST, #3501; Mouse mitochondria antibody (TOMM20) CST, #42406; Mouse lysosome antibody (LAMP1) CST, #9091; Mouse Golgi Apparatus antibody (RCAS1) CST, #12290; Human Anti-CD3 BUV395 (clone SK7) BD Biosciences, 564001; Human CD4 FITC (clone RPA-T4) BD Biosciences, 555346; Human CD3 BUV395 (clone SK7) BD Biosciences, 564001; Human CD8 PerCP-Cy5 (clone RPA-T4) BD Biosciences,565310; Human IFN-γ PE (clone B27) BD Biosciences, 559327; Human TNF-α APC (clone MAb11) BD Biosciences, 554514; Human IL-2 APC-R700 (clone MQ1-17H12) BD Biosciences, 565136; Human CD45RO PE-Cy™7 (clone UCHL1) Biolegend, 304230; Human Anti-PD-1 BV650 (clone MIH4) BD Biosciences, 564324; live/dead FVS780 BD Biosciences, 565388; Human IFN-γ Flex Set Bead B8 BD Biosciences, 565388, 560111.

All antibodies and tetramers were tested to identify working dilutions for optimal staining prior to use in this study.

Immunofluorescence staining: Using FITC-labelled OVA as a model antigen, inguinal LNs were acquired at 48 hours following PNVAC injection. For colocalization studies with DCs and T cells, 8-μm frozen sections of the LNs were stained with anti-CD11c and anti-CD3 antibodies. The sections were blocked using PBS supplemented with 10% goat serum for 1 hour at room temperature in the dark and stained with anti-CD3 antibody (Abcam, United Kingdom) and anti-CD11c rabbit monoclonal antibody (Cell Signaling

Technology, United States) overnight at 4°C. After washing with PBS 3 times, the sections were stained with goat anti-rabbit IgG H&L (Cy3) (Abcam, United Kingdom), goat anti-rat IgG H&L (Cy5) (Abcam, United Kingdom) and DAPI (Sangon Biotech, China). Immunofluorescence images were obtained using a confocal microscope (Leica, Germany).

In vitro uptake assay: Bone marrow-derived dendritic cells (BMDCs) were generated by culturing bone marrow cells flushed from femurs of 615-strain mice in DC media: DMEM supplemented with 10% FBS, pen/strep, sodium pyruvate, 20 ng/mL GM-CSF (Peprotech) and 10 ng/mL IL-4 (Peprotech). Media was replaced on day 3; non- adherent and loosely adherent immature dendritic cells (iDCs, routinely 60-80% CD11c+) were collected on day 6. iDCs were incubated with NS, free neoantigens (MFC-1 or MFC-2, to MFC-9) and neoantigen-based nanovaccines for 48 hours and phenotyped by determining the expressions of CD11c, CD86 and CD80. Cy5 labeled free neoantigens or PNVAC (1 μg/mL per peptide) was incubated with iDCs at 37°C for 1 min, 20 min, 40 min, 60 min and 120 min and imaged using a total internal reflection fluorescent microscope (Leica Thunder Imager). In some experiments, iDCs were firstly incubated with one of the four inhibitors: micropinocytosis inhibitor LY294002 (Sigma, L9908, 20 μM), clathrin inhibitor PitoStop2 (Abcam, ab120687, 10 μM), caveolin inhibitor WL-47 (MCE, HY-P2288, 500 nM), dynamin inhibitor dynasore (Sigma, D7693, 5.7 μM) for 2 hours. Then washed iDCs were added with Cy5 labeled free neoantigens or PNVAC (1 μg/ml per peptide) and imaged using a total internal reflection fluorescent microscope after 120 min.

Organelle distribution: iDCs were incubated with Cy5 labeled free neoantigens or PNVAC (1 μg/ml per peptide) for 2 hours and then fixated with 4 % paraformaldehyde (Sigma-Aldrich, USA) for 10 min at room temperature. After that, DCs were incubated with one of the five organelle antibodies: tubulin antibody (Abcam, ab7291, 1:200), Endoplasmic reticulum antibody (CST, #3501, 1:200), mitochondria antibody (CST, #42406, 1:400), lysosome antibody (CST, #9091, 1:200), Golgi Apparatus antibody (CST, #12290, 1:200), overnight at 4 ℃, followed by incubation with secondary antibodies, Plasma membrane antibody (iFluor 488-Wheat Germ Agglutinin conjugate, AAT Bioquest, #25530） and DAPI. Fluorescence images were acquired by Leica Thunder Imager and analyzed by LAS X software.

Preparation of tissues and cells: Peripheral blood samples were collected from the orbital sinus. Spleens, LNs, and tumors were stored in NS. Single-cell suspensions were

prepared in NS from spleens and LNs by grinding the tissue with the frosted surfaces of glass slides. Erythrocytes were removed with red blood cell lysis buffer. Tumors were cut into small pieces, digested with collagenase IV (1 mg mL-1; Sigma), and passed through cell strainers. Bone marrow DCs were flushed from the femur and tibia, homogenized and filtered, followed by erythrocytes removal with red blood cell lysis buffer.

Flow cytometry: All samples tested were stained with indicated extracellular antibodies for 20 min at 4°C in the dark, and washed before analysis. Flow cytometric data were acquired using a BD Accuri C6 cytometer (BD Biosciences, United States) and analyzed using FlowJo v10 software (TreeStar, United States). The BD™ CBA Mouse Th1/Th2 Cytokine Kit, IL-6 Flex Set, and IL-10 Flex Set were used to detect multiple cytokines (IL-2, IL-4, IL-5, IFNγ, TNFα, IL-6, and IL-10).

In vitro cytotoxicity assay: Freshly isolated splenocytes were tested using a carboxyfluorescein succinimidyl ester (CFSE)/propidium iodide (PI) labelling cytotoxicity assay. MFC gastric cancer cells were stained with CFSE (Invitrogen, United States) for 10 minutes at 37°C in the dark. Splenocytes from mice in each group were then incubated with CFSE-labelled MFC cancer cells at E:T ratios of 12.5:1, 25:1, and 50:1 at 37°C and 5% CO2. Six hours later, PI was added to the mixed cells, which were then incubated for 20 minutes at 4°C in the dark. Flow cytometry was performed to examine the proportion of dead tumor cells.

IFN-γ ELISPOT assay: An IFN-γ ELISPOT kit (Dakewei, China) was used to evaluate the cytokine secretion of T cells after overnight activation with peptides. Briefly, freshly isolated splenocytes from 615-strain mice in each group (NS, free vaccines, nanovaccines, 1×105 per well, 100 μl AIM-V medium containing 10% FCS per well) were added to wells with peptides (50 μg ml-1) in triplicate and incubated for 18 hours at 37°C. The cells were then lysed with cold water and the wells were washed before sequential incubations with biotinylated anti-IFN-γ antibody and streptavidin-AP for 1 hour at 37°C. A 3-amino-9-ethylcarbazole (AEC) solution mixture was then added and the plates were incubated in the dark for approximately 25 minutes at 37°C. Finally, all wells were scanned and analyzed using an ELISPOT CTL Reader (Cell Technology Inc., Columbia, MD, United States).

Analysis of changes in gene expression: Total RNA was extracted from NS- or nanovaccine-treated tumors using Trizol Reagent (Qiagen, China). The concentration and quality of the isolated total RNA were determined by an Agilent 2100/2200 Bioanalyzer (Agilent Technologies, Palo Alto, CA, United States) and a NanoDrop

(Thermo Fisher Scientific Inc.). One microgram of total RNA was used for mRNA sequencing on an Illumina HiSeq/NovaSeq or MGI2000 (Genewiz, China). Genes expressed differentially between the NS- and nanovaccine-treated tumors were detected using HTSeq (v0.6.1) and the DESeq2 Bioconductor package (fold change ≥ 1.5 and $P < 0.05$) and plotted on a heat map. Ingenuity pathway analysis software (Qiagen, China) was used to explore the expression of the gene pathways of immunity. The associations of some differentially expressed genes with type Ⅰ IFN responses and the MHC Ⅰ and MHC Ⅱ pathways were analyzed by Gene Ontology and Kyoto Encyclopedia of Genes and Genomes enrichment analyses. Their expressions in tumor-infiltrating DCs were summarized from RNA-seq data and plotted on a heat map.

Generation of personalized neoantigens

WES: Paired blood and tumor tissue were collected from patients and gDNA was extracted for library preparation. For gDNA extraction, formalin-fixed paraffin-embedded (FFPE) tumor sections and corresponding blood were processed. Then, 50–250 ng double-stranded DNA was fragmented to ~250 bp using sonication, followed by library construction with a KAPA Hyper Prep Kit (KAPA Biosystems). Probes designed at OrigiMed, Shanghai, covering the exonic regions of more than 20,000 human coding genes, as well as introns with high pathogenic fusion frequency, were used to detect mutations. Sequencing was performed using an Illumina NovaSeq 6000 (Illumina Inc., United States) with a mean depth of 500× at the OrigiMed, a laboratory certified by the College of American Pathologists and Clinical Laboratory Improvement Amendments “YuanSu” panel. For patients 002, 003, 005, and 041, nonsynonymous mutations were identified using an OrigiMed custom hybridization capture panel, which encodes exons of 450 key cancer-related genes and selected introns of 36 genes commonly rearranged in solid tumors. The probe density was increased to ensure capture efficiency in the conservatively low-read depth region. Targeted library fragments were captured according to the protocol of hybridization capture of DNA libraries using xGen Lockdown Probes and Reagents (Integrated DNA Technologies, San Diego, CA, United States). Post-capture libraries were sequenced with a mean coverage of 900× for FFPE samples and 300× for matched blood samples on an Illumina NovaSeq 6000 platform (Illumina Inc., United States).

RNA-seq: RNA was extracted from unstained FFPE sections using the miRNeasy FFPE Kit (cat# 217504, Qiagen) according to the manufacturer’s protocol. The yield and quality of extracted RNA were assessed using the Qubit™ RNA HS Assay Kit

(ThermoFisher Scientific) and LabChip GX Touch HT Nucleic Acid Analyzer (Perkin Elmer), respectively. After ribosomal RNA depletion by the NEBNex rRNA Depletion Kit (cat# E6310L, New England Biolabs), cDNA synthesis was performed using M-MLV RT RNase (H-) (cat# M3683, Promega) and an NEB Second Strand mRNA synthesis kit (cat#E6111L, New England Biolabs). Sample library preparation was performed using a KAPA Hyper Prep Kit (KAPA Biosystems) and sequenced on a NovaSeq 6000 platform with 2×151 bp paired-end reads according to the manufacturer's instructions. The relative abundance of each annotated transcript was reported as transcripts-per-million and log2-transformed before analysis. Sequencing was performed and analyzed by OrigiMed.+-

Prediction and identification of target epitopes: OptiType was used to identify class I HLA (HLA-A, HLA-B, and HLA-C) alleles and class Ⅱ HLA-DRB1 alleles. After all nonsynonymous mutations were identified using next generation sequencing (NGS), the mutant epitopes were predicted using NetMHCpan v3.1 and NetMHCIIpan v1.2 tools as described previously [3]. Substrings within the 15 mers that had a binding affinity of less than 500 nM or % rank < 2.0 for any patient's HLA allele were considered to be candidates and peptides with strong hydrophobicity were excluded.

Cytometric Bead Array analysis of cytokines: The concentrations of IFN-γ in culture supernatants for ex vivo and in vitro stimulation study were measured using CBA according to the manufacturer's protocol (BD Biosciences, United States). The samples were subjected to a CytoFLEX LX flow cytometer (Beckman Coulter, United States) and the data were analyzed. The IFN-γ of mutant peptide-stimulated PBMCs being more than double that of no-peptide control was identified as indicating positive PBMCs reactivity.

IFN-γ ELISPOT assay: For in vitro pre-stimulated PBMCs, the secretion of IFN-γ released from T cells was evaluated using an IFN-γ ELISPOT kit (Dakewei, China) after overnight stimulation with irradiated autogenous PBMCs loaded with a corresponding peptide [1]. Briefly, pre-stimulated PBMCs (105 per well) with irradiated autogenous PBMC loaded with a corresponding peptide (25 μg mL-1) were added to wells in triplicate for 18-20 hours of incubation in AIM-V culture medium. After washing, the diluted detection antibody was added for 1 hour of incubation at 37°C. Streptavidin-HRP (1:100 dilution) and 3-Amino-9-ethylcarbazole solution mixture were then added to each well in turn. After the plates were kept in the dark for approximately 20 minutes at room temperature, deionized water was added to stop the reaction. The plates were scanned under an ELISPOT CTL Reader (Cellular Technology Inc.) and the data were analyzed

using ELISPOT software (AID). The positive PBMC reactivity was scored when spots were more than twice the size of the negative control.

Intracellular cytokine staining: In vitro multiparameter ICS assays were performed as previously described [2]. Briefly, frozen PBMCs were thawed and cultured in media supplement with cytokines for 11 days of stimulation, ICS was then performed. A total of 1×10^6 antigenic peptide-stimulated PBMCs per well were re-stimulated with irradiated autogenous PBMC at an E: T of 2: 1, and pooled peptides (each at 1.5–2.0 μg ml-1) were added to the culture. The T cells were treated with GolgiStop (BD Biosciences) according to the manufacturer's protocols for 8 hours the following day. The stimulated T cells were then stained for 30 minutes at room temperature with a fixable live/dead stain (FVS780), anti-CD3 (BUV395), anti-CD4 (FITC), anti-CD8 (PerCP-Cy™5.5), anti-PD-1 (BV650, BD Biosciences, United States) and anti- CD45RO (PE-Cy™7) antibodies (BioLegend, United States). A Fixation/Permeabilization Solution Kit was used to fix and permeabilize the cells (BD Biosciences, United States). After that, anti-IFN-γ (PE), anti-IL-2 (APC-R700) and anti-TNF-α (APC) antibodies (BD Biosciences) were added to stain the cells in order to measure intracellular cytokines at 4 °C. The cells were washed with permeabilization buffer and fixed with 1% paraformaldehyde solution (Sigma-Aldrich). Flow cytometry was performed and the data were analyzed using FlowJo v10 software. Live and dead lymphocytes were stained and CD3+ T cells were gated. CD4+ and CD8+ T cells were separately gated and cytokine staining was plotted for each population (Supplementary Fig. 10).

Reference

1. F. Chen, Z. Zou, J. Du, S. Su, J. Shao, F. Meng, J. Yang, Q. Xu, N. Ding, Y. Yang, Q. Liu, Q. Wang, Z. Sun, S. Zhou, S. Du, J. Wei, B. Liu, Neoantigen identification strategies enable personalized immunotherapy in refractory solid tumors. J Clin Invest 129, 2056-2070 (2019).

2. P. A. Ott, Z. Hu, D. B. Keskin, S. A. Shukla, J. Sun, D. J. Bozym, W. Zhang, A. Luoma, A. Giobbie-Hurder, L. Peter, C. Chen, O. Olive, T. A. Carter, S. Li, D. J. Lieb, T. Eisenhaure, E. Gjini, J. Stevens, W. J. Lane, I. Javeri, K. Nellaiappan, A. M. Salazar, H. Daley, M. Seaman, E. I. Buchbinder, C. H. Yoon, M. Harden, N. Lennon, S. Gabriel, S. J. Rodig, D. H. Barouch, J. C. Aster, G. Getz, K. Wucherpfennig, D. Neuberg, J. Ritz, E. S. Lander, E. F. Fritsch, N. Hacohen, C. J. Wu, An immunogenic personal neoantigen vaccine for patients with melanoma. Nature 547, 217-221 (2017).

a b

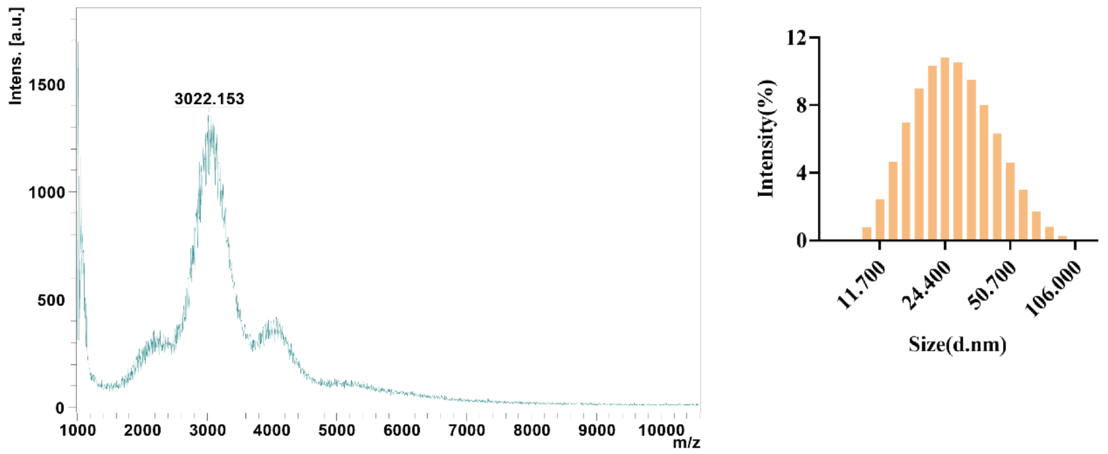

c

Nanoparticles (NP)	Encapsulation efficiency (%)	Drug loading content (%)	Size (nm)	PDI	Zeta-potential
MFC-1-NP	34.83±1.80	11.61±0.60			
MFC-2-NP	38.34±0.59	12.78±0.20			
MFC-3-NP	29.52±1.34	9.84±0.45			
MFC-4-NP	44.61±4.11	14.87±1.37			
MFC-5-NP	22.8±0.50	7.60±0.17			
MFC-6-NP	33.7±0.62	11.23±0.21			
MFC-7-NP	26.50±0.55	8.83±0.18			
MFC-8-NP	22.67±2.89	7.56±0.96			
MFC-9-NP	32.28±0.84	10.76±0.28			
PNVAC(mix)			21.94±2.88	0.29±0.01	-27.80±0.89

d

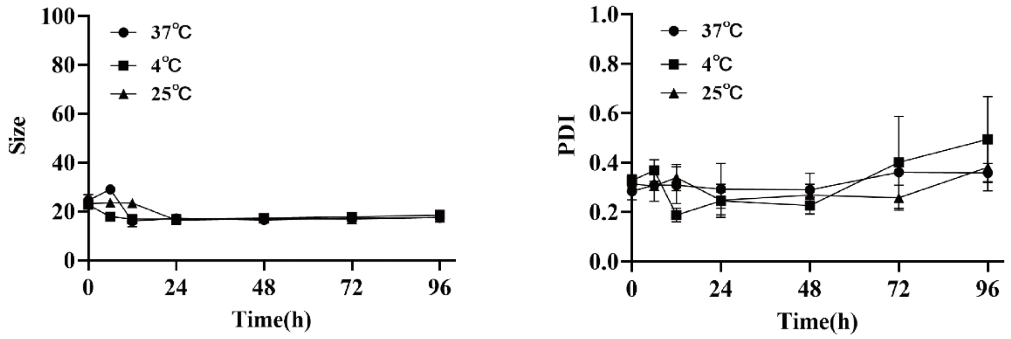

e f

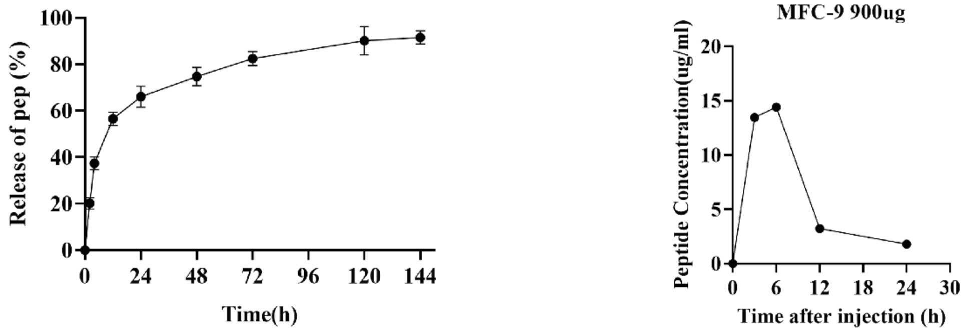

Figure S1. Characterization of Personalized neoantigen nanovaccines (PNVAC). (a) Molecular weight characterization of DSPE-PEG$_{2000}$-Peptide analyzed using MALDI-TOF mass spectrometry. (b) Size of PNVAC analyzed using a Malvern Zetasizer. (c) Encapsulation efficiency, drug loading content of nine neoantigen-based nanoparticles (MFC-1-NP, MFC-2-NP to MFC-9-NP) and size, polydispersity index (PDI) and zeta potential of PNVAC (mix of nine neoantigen-based nanoparticles). (*n* = 3, mean ± s.d). (d) Size and PDI of PNVAC were kept stable when nanovaccines were incubated

in normal saline for 96 h at 4℃, 25℃ or 37℃ (n = 3, mean ± s.d). (e) Curves of peptide (MFC-9) release from MFC-9-NP at room temperature (about 25℃). (f) 615-strain mice were injected subcutaneously with 900 μg PNVAC mixed with Montanide ISA^{TM} 51 at the tail base. MFC-9 peptide concentration in blood was quantified by HPLC.

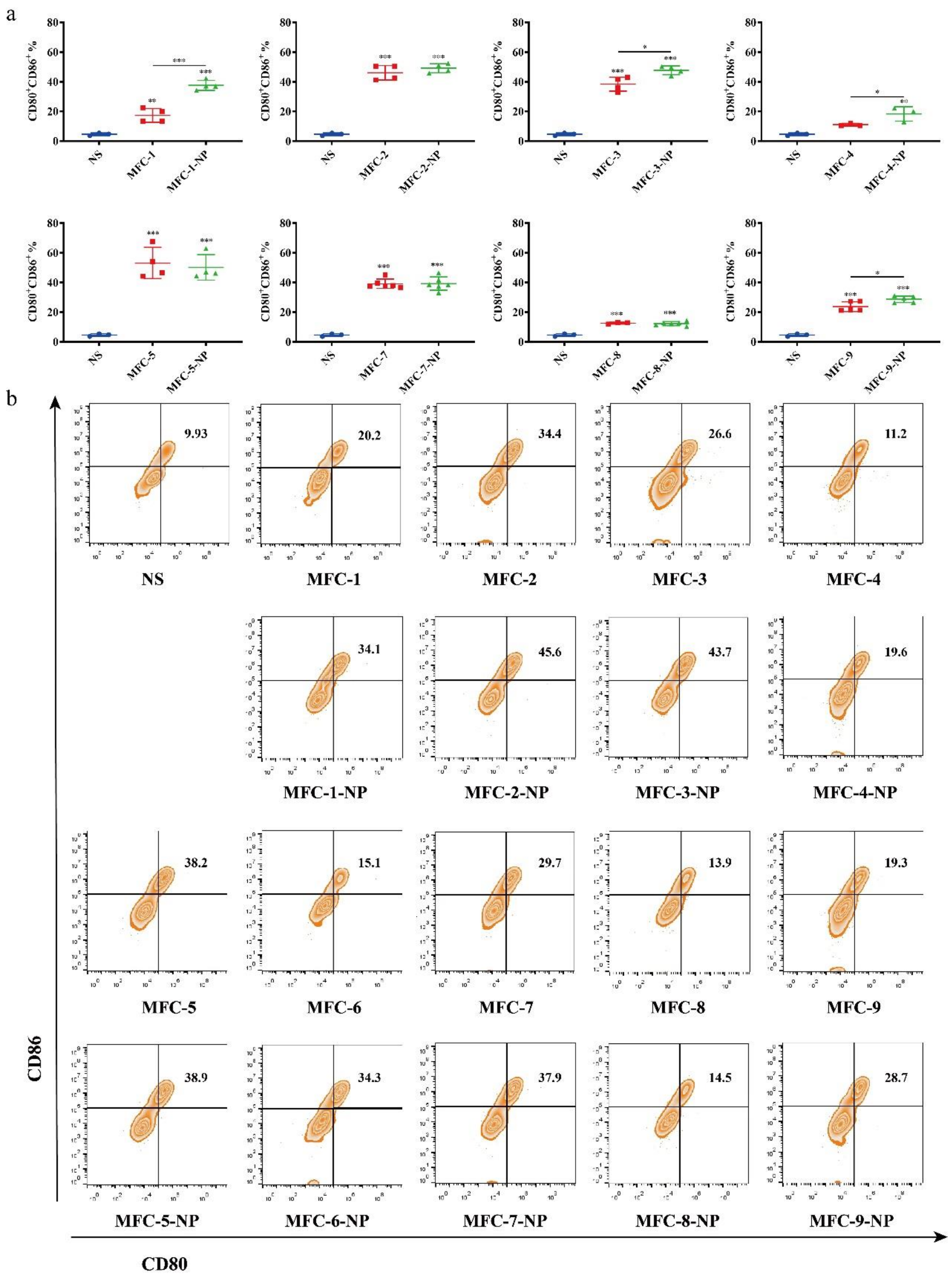

Figure S2. Personalized neoantigen nanovaccines (PNVAC) activate bone marrow-derived dendritic cells (BMDCs). Statistical data (a) and representative flow cytometry analysis (b) showed frequency of mature DCs (CD11c$^+$CD80$^+$CD86$^+$ DCs) after in vitro incubation BMDCs with normal saline (NS), free neoantigens (MFC-1 or MFC-2 to MFC-9) and neoantigen-based nanovaccines for 48 hours (n = 4,

mean ± s.d). P-values were determined by one-way ANOVA with Tukey's multiple comparisons test. ***$P < 0.001$, **$P = 0.0027$, *$P = 0.0488$.

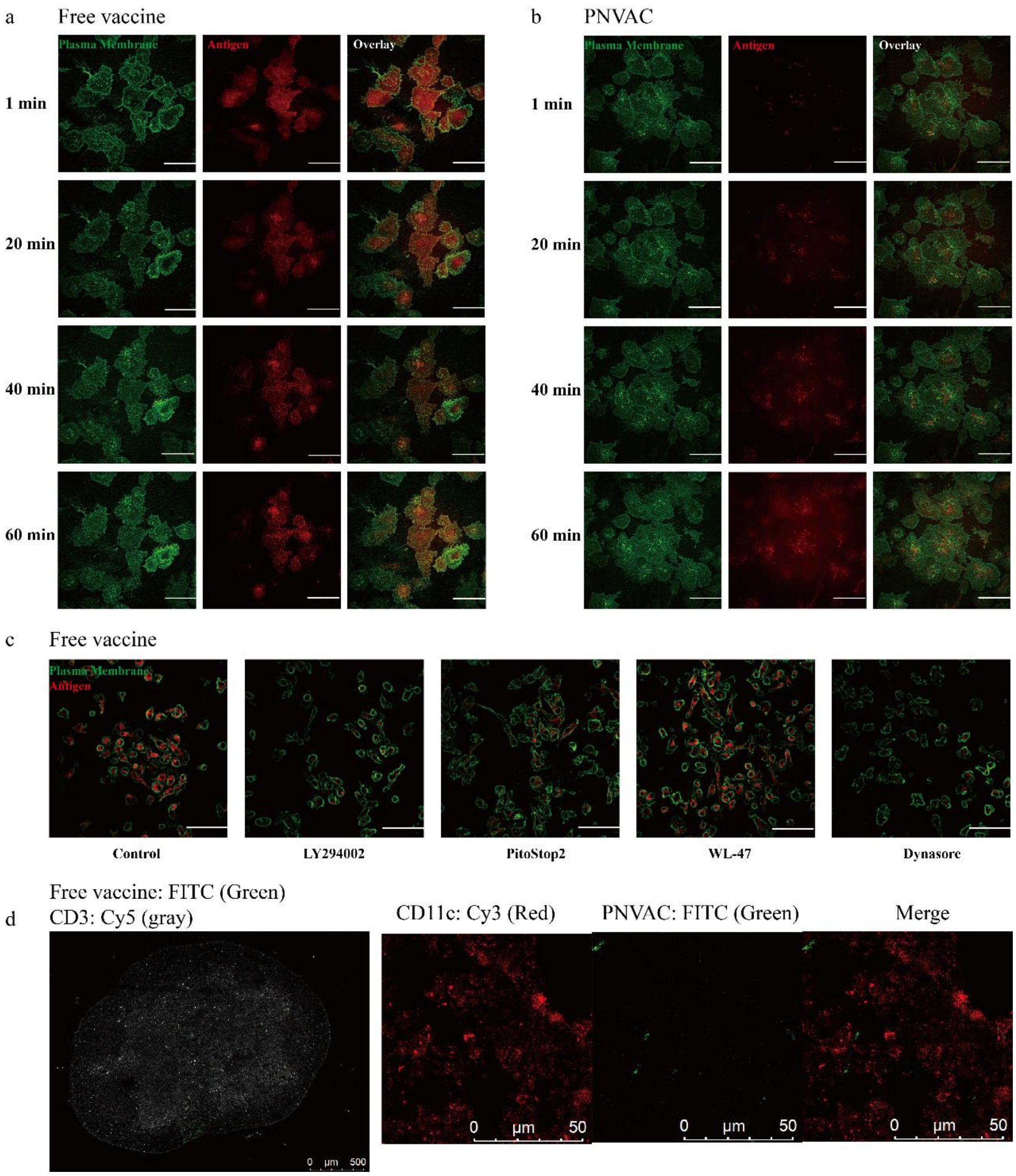

Figure S3. In vitro uptake of personalized neoantigen nanovaccines (PNVAC) by bone marrow-derived dendritic cells (BMDCs). After Cy5-labeled free vaccine (a, green) or PNVAC (b, green) were added into BMDCs (plasma membrane: red), fluorescence images were taken at 1 min, 20 min, 40 min, 60 min by total internal

reflection fluorescent microscope. Scale bar, 40 μm. (c) Normal saline (NS, control) and four inhibitors were separately added into BMDCs (plasma membrane: red) before incubation with free vaccine (green). Fluorescence images were taken at 60 min after incubation by total internal reflection fluorescent microscope. Scale bar, 30 μm. micropinocytosis inhibitor: LY294002, clathrin inhibitor: PitoStop2, caveolin inhibitor: WL-47, dynamin inhibitor: dynasore. (d) Localization of $CD3^+$ T cells (gray) or $CD11c^+$ DCs (red) and FITC-labeled free vaccine (green) in LNs at 48 hours after subcutaneous injection. Scale bar, 500 μm, 50 μm.

a

b

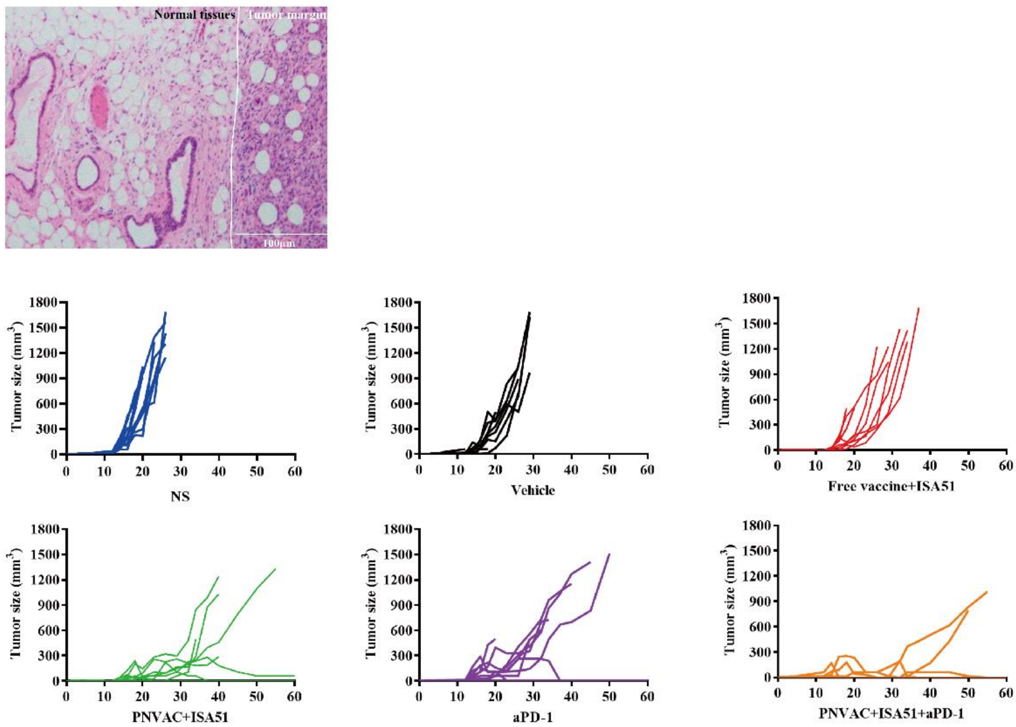

Figure S4. Personalized neoantigen nanovaccines (PNVAC) inhibit tumor growth. (a) Hematoxylin and eosin staining of tissues sections from margins of tumor dissection (scale bar, 100 μm). (b) MFC cells (2×10^6 cell per mouse) were subcutaneously injected into 615-strain mice. The established tumors were removed when the tumor volume reached ~100 mm^3. Three days after surgery, the tumor-bearing mice were randomly divided into 6 groups and injected

subcutaneously with NS (normal saline), vehicle, free vaccines+ISA 51 (20 μg per peptide, 50 μl ISA 51 per mouse), PNVAC+ISA 51, aPD-1 (anti-PD-1, 0.5 mg/kg, intraperitoneally injected) or PNVAC+ISA 51+aPD-1. Mice were immunized with PNVAC five times on the indicated days. Shown are individual MFC tumor growth curves in different groups (n = 10).

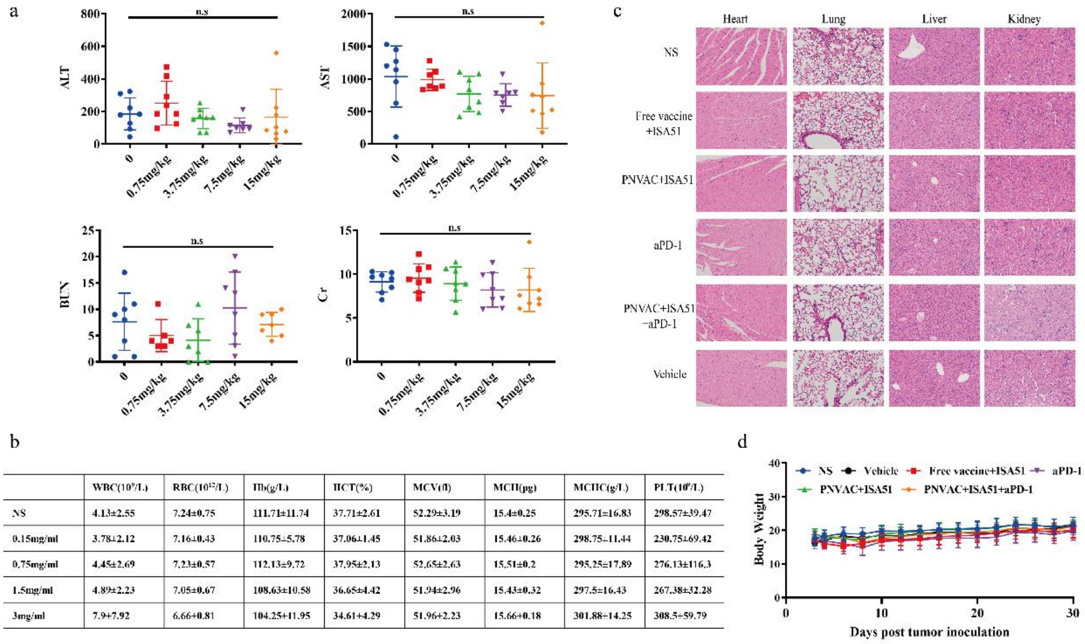

	WBC(10^9/L)	RBC(10^{12}/L)	Hb(g/L)	HCT(%)	MCV(fl)	MCH(pg)	MCHC(g/L)	PLT(10^9/L)
NS	4.13±2.55	7.24±0.75	111.71±11.74	37.71±2.61	52.29±3.19	15.4±0.25	295.71±16.83	298.57±39.47
0.15mg/ml	3.78±2.12	7.16±0.43	110.75±5.78	37.06±1.45	51.86±2.03	15.46±0.26	298.75±11.44	230.75±69.42
0.75mg/ml	4.45±2.69	7.23±0.57	112.13±9.72	37.95±2.13	52.65±2.63	15.51±0.2	295.25±17.89	276.13±116.3
1.5mg/ml	4.89±2.23	7.05±0.67	108.63±10.58	36.65±4.42	51.94±2.96	15.43±0.32	297.5±16.43	267.38±32.28
3mg/ml	7.9±7.92	6.66±0.81	104.25±11.95	34.61±4.29	51.96±2.23	15.66±0.18	301.88±14.25	308.5±59.79

Figure S5. Safety of personalized neoantigen nanovaccines (PNVAC). (a-b) A total of 615-strain mice were randomly divided into 5 groups and subcutaneously injected with NS and 4 doses of PNVAC (0.75 mg/kg, 3.75 mg/kg, 7.5 mg/kg and 15 mg/kg). Shown are hematology indices (n = 8). P-values were determined by one-way ANOVA with Tukey's multiple comparisons test. n.s not significant. (c-d) MFC cells (2×10^6 cell per mouse) were subcutaneously injected into 615-strain mice. The established tumors were removed when the tumor volume reached ~100 mm^3. Three days after surgery, the tumor-bearing mice were randomly divided into 6 groups and injected subcutaneously with NS (normal saline), vehicle, free vaccines+ISA 51 (20 μg per peptide, 50 μl Montanide ISA™ 51 per mouse), PNVAC+ISA 51, aPD-1 (anti-PD-1, 0.5 mg/kg, intraperitoneally injected) or

PNVAC+ISA 51+ aPD-1. (c) Hematoxylin and eosin staining of tissues sections from main organs, including heart, lung, liver and kidney (scale bar, 100 μm). (d) Body weights of MFC tumor bearing mice in each group (n = 4).

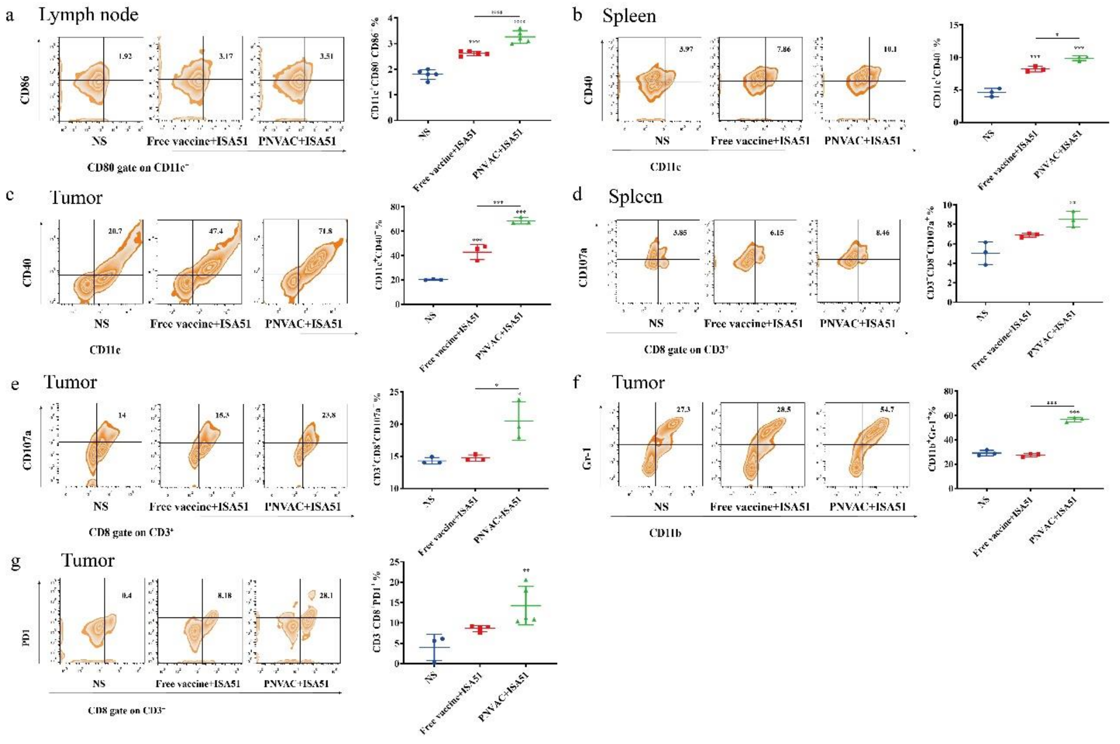

Figure S6. Personalized neoantigen nanovaccines (PNVAC) induce DCs mature and improve tumor microenvironment. (a) Proportions of CD11c$^+$CD80$^+$CD86$^+$ DCs in lymph nodes were analyzed using flow cytometry one week after the last vaccination. P-values were determined by one-way ANOVA with Tukey's multiple comparisons test. ***P < 0.001. (b-c) Proportions of CD11c$^+$CD40$^+$ DCs in spleens and tumors were analyzed using flow cytometry one week after the last vaccination. P-values were determined by one-way ANOVA with Tukey's multiple comparisons test. ***P < 0.001, *P = 0.0128. (d-e) Proportions of CD3$^+$CD8$^+$CD107$^+$ T cells in spleens and tumors were analyzed using flow cytometry one week after the last vaccination. P-values were determined by one-way ANOVA with Tukey's multiple comparisons test. **P = 0.0048, *P = 0.0128 (NS vs Free vaccine+ISA51), *P = 0.0177 (free vaccine+ISA 51 vs PNVAC+ISA51). (f) Proportions of CD11b$^+$Gr-1$^+$ NK

cells in tumors were analyzed using flow cytometry one week after the last vaccination. P- values were determined by one-way ANOVA with Tukey's multiple comparisons test. ***P < 0.001. (g) PNVAC increased PD-1^{+}CD8^{+} T cells in tumors. P-values were determined by one-way ANOVA with Tukey's multiple comparisons test. **P = 0.008.

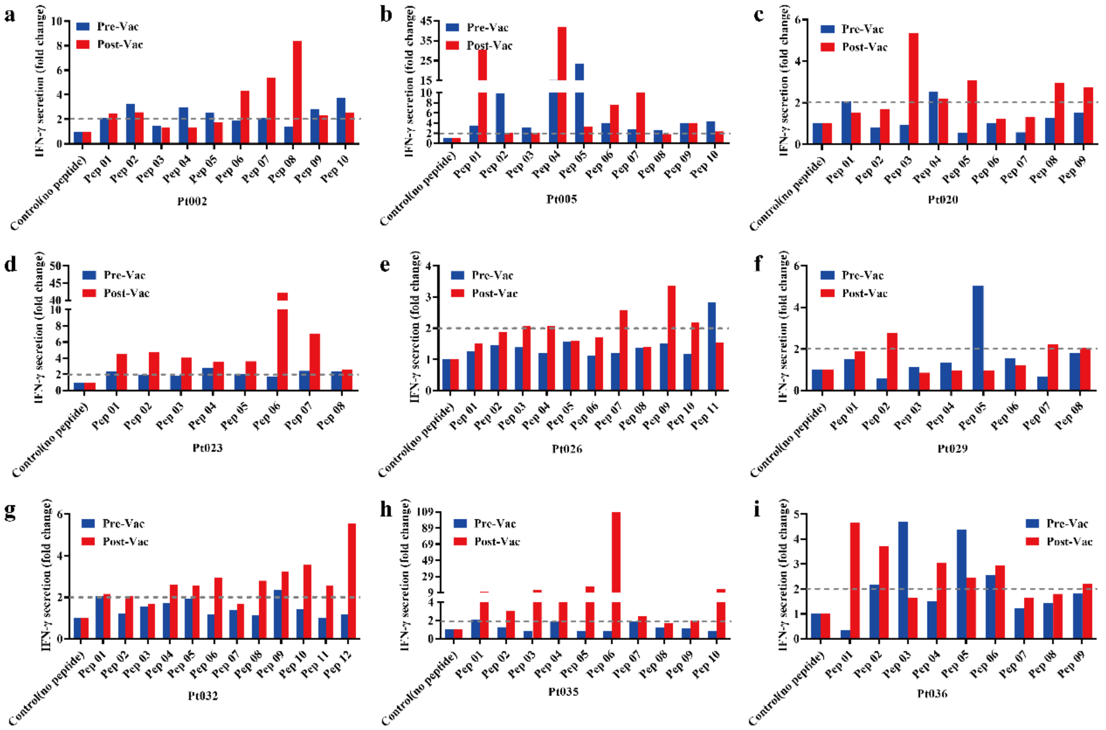

Figure S7. Demonstration of epitope spreading. IFN-γ secretion by PBMCs pre- and post- vaccination followed by overnight incubation with individual peptides or no peptide was assessed using ex vivo CBA assay.

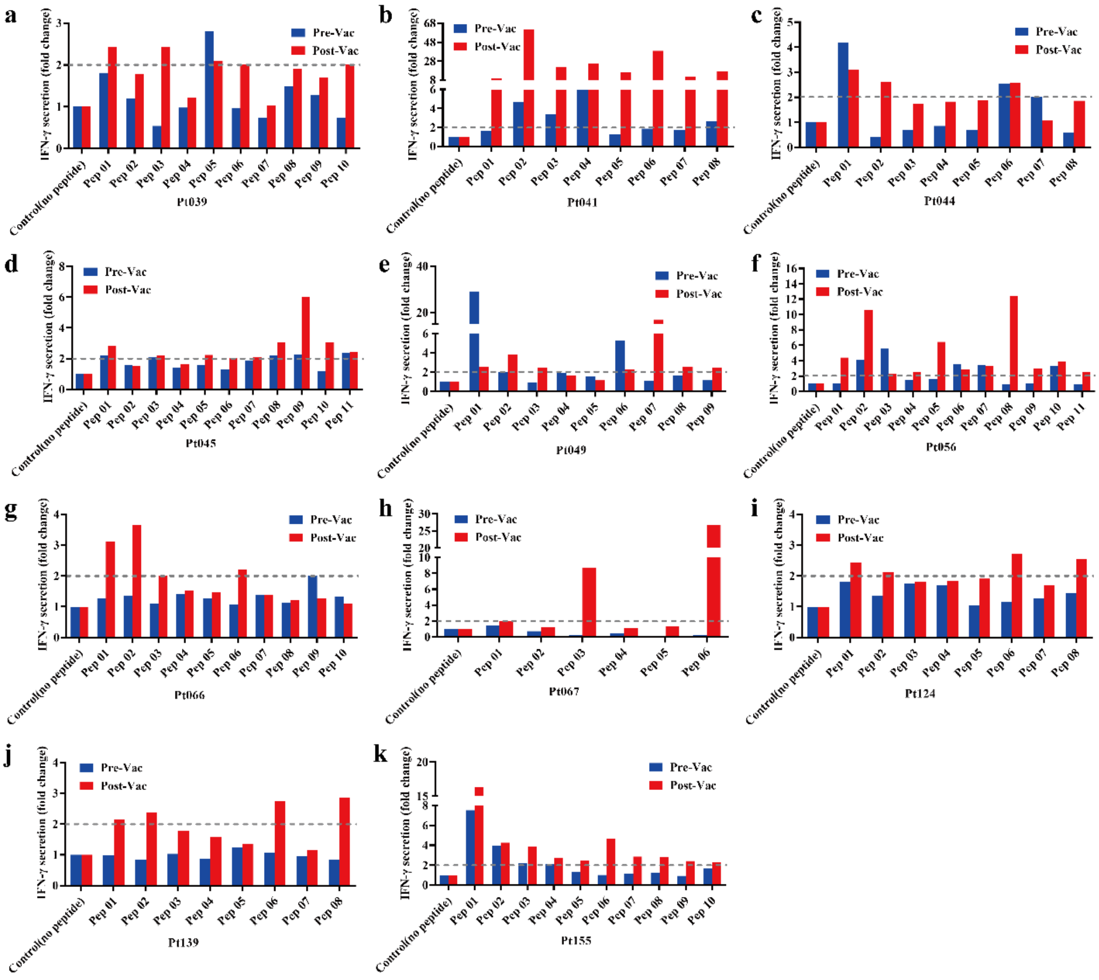

Figure S8. Demonstration of epitope spreading. IFN-γ secretion by PBMCs pre- and post- vaccination followed by overnight incubation with individual peptides or no peptide was assessed using ex vivo CBA assay.

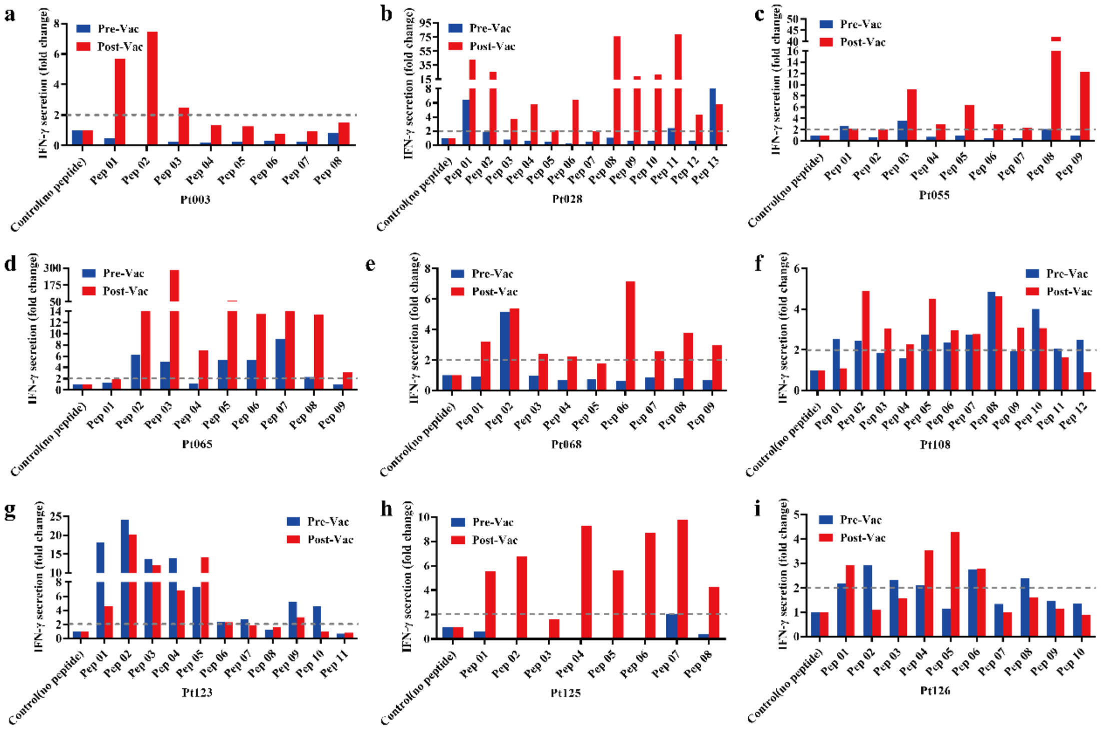

Figure S9. Demonstration of epitope spreading. IFN-γ secretion by PBMCs pre- and post- vaccination followed by 11-day stimulation with individual peptides or no peptide was assessed using in vitro CBA assay.

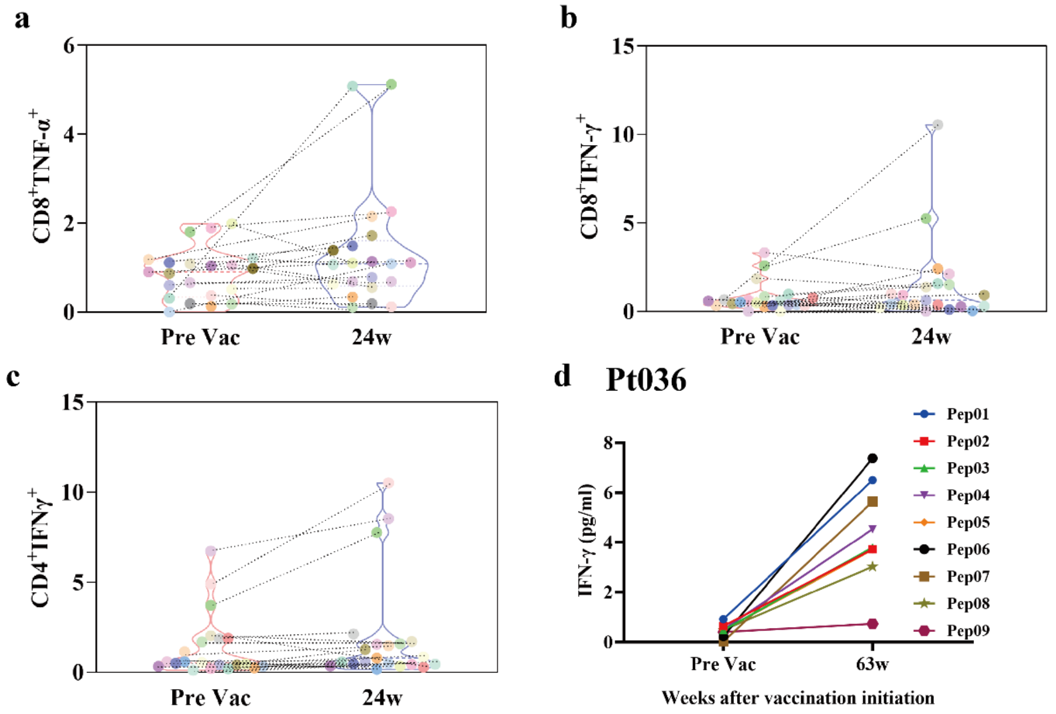

Figure S10. The neoantigen specific T cell responses. (a-c) Frequencies of cytokine-producing CD8$^+$ and CD4$^+$ T cells in response to neoantigens in 21 patients, as measured by ICS after *in vitro* stimulation of PBMCs with the immunizing peptides before and after PNVAC treatment. Data was analyzed by paired two-tailed Student's t-test. (d) The PBMCs of patient 036 at different time points were restimulated with immunized peptides overnight. Then the IFN-γ concentration in culture supernatant was measured by IFN-γ CBA assay.

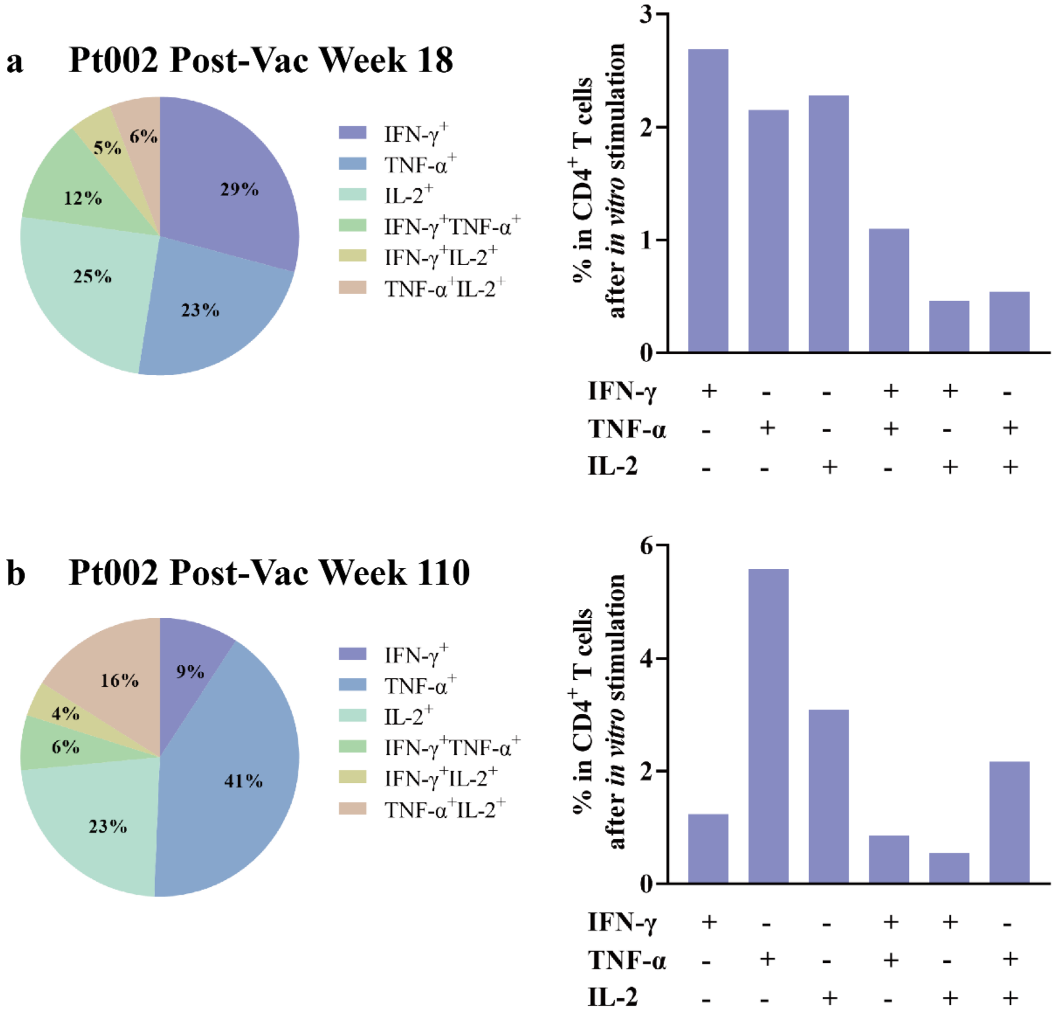

Figure S11. CD4$^+$ T cell responses in patient 002. Total CD4$^+$ T cell responses positive for one or two cytokines at week 18 and week 110 post vaccination. Bar

graphs show absolute frequencies of immunized neoepitopes-specific $CD4^+$ T cells secreting one or two cytokines.

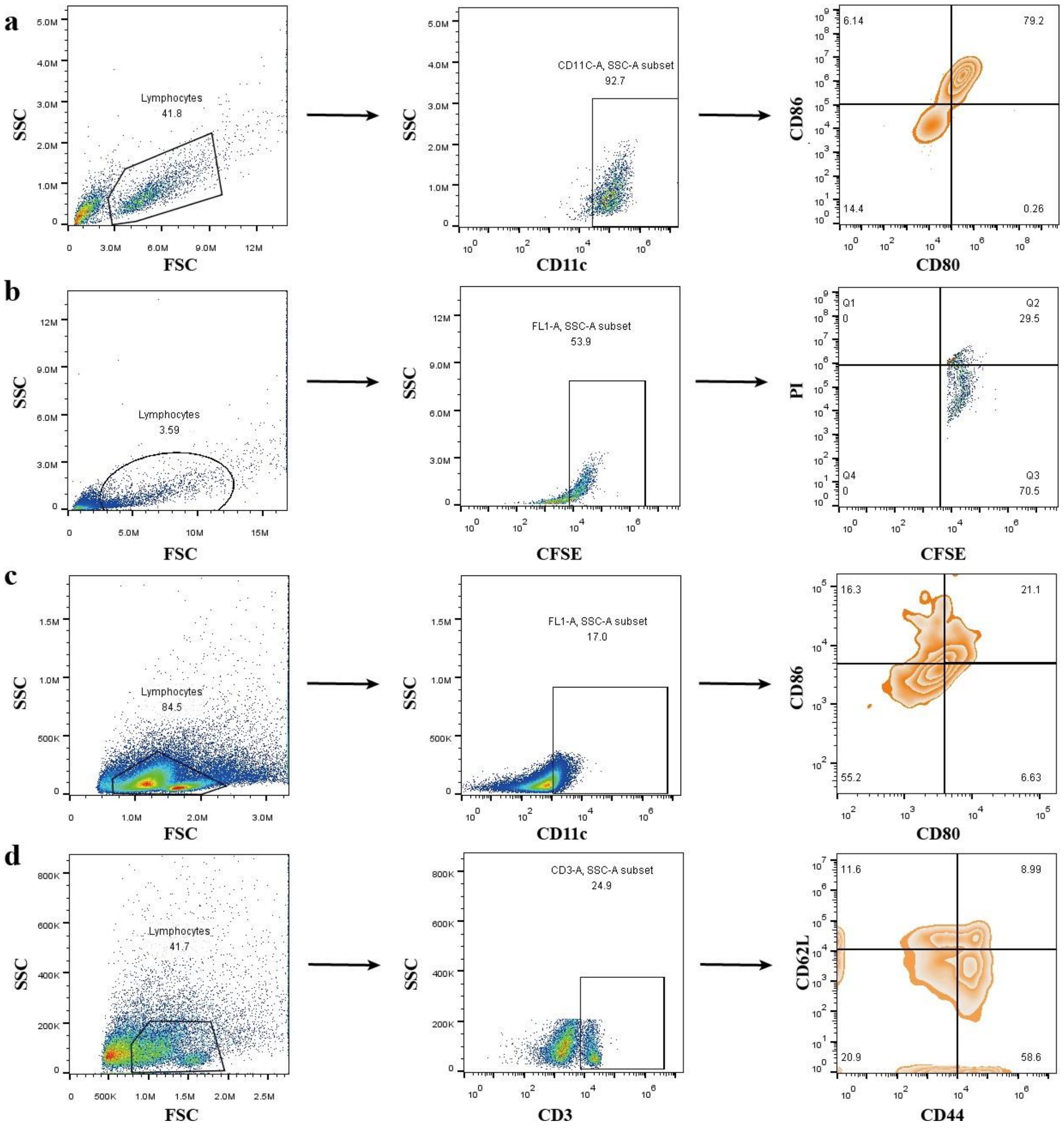

Figure S12. Gating strategy. For all stainings, a lymphocyte gate was drawn using FSC/SSC gating first. For intracellular cytokine staining, gating was determined based on negative control (no peptide stimulation).
