# Supplementary material for: Benefits of an Immunogenic Personalized Neoantigen Nanovaccine in Patients with High‐Risk Gastric/Gastroesophageal Junction Cancer
Source: Adv Sci (Weinh). 2022 Nov 9;10(1):2203298. doi: 10.1002/advs.202203298 (PMC9811442; doi:10.1002/advs.202203298)
Supplement: Supplementary file 2 — Supplemental Table 1 [file ADVS-10-2203298-s004.pdf]

Patient ID	Age	Gender	ECOG	(Gastric/ Gastroesphageal Junction (G/EGJ)	Stage	Previous Treatment	Recurrence since vaccination intiation Y/N(Weeks）
002	40	M	0	EGJ	IIIB	S-1+docetaxel	N
003	53	F	1	G	IIIB	S-1+docetaxel	N
005	46	M	0	G	IIIC	S-1+docetaxel	Y(53.7)
020	60	M	0	G	IIIB	S-1+docetaxel	N
023	66	M	1	G	IIIB	S-1+docetaxel	Y(45)
026	75	M	0	G	IIIB	S-1+oxaliplatin	N
028	57	M	1	EGJ	IIIB	S-1+oxaliplatin	N
029	45	M	0	G	IIIB	S-1+docetaxel	N
032	75	F	1	EGJ	IIIB	S-1+docetaxel	N
035	57	M	0	EGJ	IVA	S-1+docetaxel	N
036	64	M	1	EGJ	IVA	S-1+docetaxel	N
039	65	M	0	G	IIIC	S-1+docetaxel	N
041	49	M	0	G	IIIB	S-1+oxaliplatin	N
044	72	M	1	G	IIIB	S-1+docetaxel	Y(7.6)
045	46	M	0	G	IIIC	S-1+docetaxel	Y(49.6)
049	75	F	1	EGJ	IIIB	S-1+docetaxel	N
055	60	F	1	G	IIIB	S-1+docetaxel	N
056	56	F	1	G	IIIB	S-1+docetaxel	N
065	65	M	1	G	IIIB	S-1+docetaxel	N
066	34	F	1	G	IIIC	capecitabine+oxaliplatin	Y(35.7)
067	66	M	0	G	IIIB	S-1+docetaxel	N
068	56	M	0	EGJ	IIIB	S-1+oxaliplatin	N
108	54	M	0	G	IIIB	S-1+docetaxel	N
123	46	F	1	G	IIIB	capecitabine+oxaliplatin	N
124	59	M	1	G	IIIB	capecitabine+oxaliplatin	N
125	60	F	1	G	IIIB	S-1+docetaxel	N
126	46	F	1	G	IIIB	S-1+docetaxel	N
139	56	F	1	G	IIIC	S-1+docetaxel	N
155	54	M	0	G	IIIB	S-1+docetaxel	N
