# Supplementary material for: Benefits of an Immunogenic Personalized Neoantigen Nanovaccine in Patients with High‐Risk Gastric/Gastroesophageal Junction Cancer
Source: Adv Sci (Weinh). 2022 Nov 9;10(1):2203298. doi: 10.1002/advs.202203298 (PMC9811442; doi:10.1002/advs.202203298)
Supplement: Supplementary file 3 — Supplemental Table 2 [file ADVS-10-2203298-s003.pdf]

Individualized neoantigens for each patient

Patient 002

Genes	Mutations	HLA	Neoepitopes	Predicted Scores
PALB2	p.P812L	A*69:01	SVPPGTPPL	60.01
PARP4	p.V1219I	B*13:01	WQGEPQEAI	308.82
PALB2	p.P812L	A*11:01	PLIESFTFK	139.67
CTNNA1	p.Q692P	A*11:01	QVASFPEEK	40.43
FLT4	p.S694L	A*11:01	DLLEMQCLV	440.21
BAP1	p.L180F	DRB1*04:05	AFHFVSYVPITGRFF	87.14
PARP4	p.V1219I	DRB1*04:05	QEAIRNQSLLASSEW	348.00
FLT4	p.S694L	DRB1*04:05	TQNLTDLLVNVSDLL	365.56
ERBB3	p.I655_L659delinsM	A*11:01	VVMGGTFLY	13.83
ROS1	p.R1569W	A*69:01	TVWSDTSLI	57.03

Patient 003

Genes	Mutations	HLA	Neoepitopes	Predicted Scores
GNAS	p.P328L	C*14:02	RILLPGQPL	280.64
GABRA6	p.A394V	DRB1*04:04	RVPILQSTPVTPPPL	243.35
GABRA6	p.A394V	DRB1*04:04	NKVLTRVPILQSTPV	157.54
BAP1	p.V24F	DRB1*09:01	FGFKGVQVEEIYDLQ	270.40
GNAS	p.P328L	DRB1*09:01	QPQRILLPGQPLQPQ	467.34
CTAs				
IMP3		A*02:01	NLSSAEVVV	
IMP3		A*02:01	RLLVPTQFV	
DEPEC1		A*02:01	FLDLPEPLL	

Patient 005

Genes	Mutations	HLA	Neoepitopes	Predicted Scores
PIK3CG	p.H94Y	A*24:02	FYHRLGPYHF	15.08
RAD51D	p.R239W	B*40:01	RELKTLAWDL	73.55
RAD51D	p.R239W	C*03:04	LAWDLGMAV	21.05
RAD51D	p.R239W	DRB1*09:01	LKTLAWDLGMAVVVT	102.64
RAD51D	p.R239W	DRB1*15:01	ALMMQLARELKTLAW	147.45
PIK3CG	p.H94Y	DRB1*09:01	ADFYHRLGPYHFLLL	63.26
CTAs				
LY6K-177			RYCNLEGPPI	
IMP3-508			KTVNELQNL	
DEPDC1-294			EYYELFVNI	
SP17-7			THYRIPQGF	

Patient 020

Genes	Mutations	HLA	Neoepitopes	Predicted Scores
ZNF862	p.S1092del	DRB1*07:01	IQHWYLTSGRRFSHV	28.93
CSNK1G1	p.I161_Y162del	A*30:01	HSKNLRDVK	30.10
PCDHB1	p.P143L	DRB1*07:01	PLLKILESTPLGSRF	44.52
PCDHGA2	p.S758F	DRB1*07:01	HEVFLTADSRKSHLI	78.47
CSNK1G1	p.I161_Y162del	DRB1*07:01	QLLSRMEYVHSKNLR	87.09
MALRD1	p.G1577V	A*30:01	RVDKAHFRST	100.50
DVL3	p.E488K	A*30:01	TVNKITFSK	122.30
KCNV1	p.T63M	C*07:02	MRLGKLAVV	196.10
ZNF862	p.S1092del	A*30:01	SGRRFSHVY	199.40

Patient 023	Genes	Mutations	HLA	Neoepitopes	Predicted Scores
	PIK3CG	W201R	A*02:03	KLYAMHPRV	10.80
	QKI	D95Y	B*35:01	VPVKEYPYF	59.70
	LRP1B	N433T	A*02:03	YLYATNSDT	76.10
	NRG1	A221S	B*35:01	APSAFRASF	116.00
	FLT1	S496N	B*35:01	FILDADNNM	147.60
	QKI	D95Y	DRB1*07:01	PVKEYPYFNFVGRIL	179.38
	FAT4	T1034I	DRB1*07:01	EIAYIIAEGNTGDAF	198.08
	PIK3CG	W201R	DRB1*07:01	RDPKLYAMHPRVTSK	225.79

Patient 026	Genes	Mutations	HLA	Neoepitopes	Predicted Scores
	F3	P.S109del	A*02:01	YLARVFYPA	2.30
	CADM3	P.A3Pfs*50	A*02:01	HLMKQWWLV	3.60
	COQ5	P.D80del	C*03:04	YVMNDMMSL	3.60
	RNF213	P.E1454Rfs*36	A*02:01	TLMWTGWPA	3.90
	GRB10	P.Y67Tfs*119	C*03:04	MASMPAASL	4.20
	B3GALT6	P.R197Afs*246	DRB1*12:01	RGRLATLRLLPALRA	60.41
	LRRCC1	P.D940Tfs*36	DRB1*12:02	LKDKLMQLLKLIKLK	78.87
	ALDH2	P.K289Rfs*122	DRB1*12:02	RGRRWMKLSLRRSSA	71.42
	PRAC2	P.C34Wfs*92	DRB1*12:02	STPLIIMARKIKKTW	79.14
	GABBR1	P.P397Qfs*32	B*13:01	SQFSPAWAL	15.30
	TRAM1L1	P.V13Ffs*128	B*15:02	FLVFGAHSF	23.90

Patient 028	Genes	Mutations	HLA	Neoepitopes	Predicted Scores
	PRAMEF2	p.C235Qfs*14	DRB1*09:01	TLQTRFLQVPSLHVR	28.50
	KIRREL3	p.A133T	A*11:01	ATIRSRPAR	86.80
	SEC63	p.F16del	B*15:01	NTFYFLTSF	110.60
	SEC63	p.F16del	DRB1*09:01	SGNTFYFLTSFVGLI	113.32
	PRAMEF2	p.C235Qfs*14	B*15:01	KEMKTLQTRF	122.20
	CDK5R2	p.A256Cfs*106	A*11:01	TPTSSRRSFK	176.00
	JMY	p.R872W	A*11:01	LVSAWKKLRK	177.00
	DTX1	p.T163M	A*11:01	SQMNRQMRR	179.60
	FTMT	p.S15L	B*15:01	LLSRHISPL	180.30
	CDK5R2	p.A256Cfs*106	DRB1*09:01	SSRRSFKTSRTRARP	209.94
	TNFRSF19	p.T125M	B*15:01	KMKLVGFQDM	262.90
	OR2A12	p.S264R	DRB1*14:05	DVKAAITKIMSQDLG	293.35
	JMY	p.R872W	DRB1*09:01	LFDSSQLVSAWKKLR	317.21

Patient 029	Genes	Mutations	HLA	Neoepitopes	Predicted Scores
	PHOX2B	p.S207Qfs*153	A*30:01	RQRPIFAPK	3.50
	TCERG1	p.K936Rfs*17	A*11:01	STLGNFWMK	4.00
	CDC25C	p.K244Sfs*15	A*30:01	KVKKSIFLA	4.60
	ZNF579	p.G437Afs*55	A*30:01	RTRAPAARA	4.60
	PGBD5	p.H227Qfs*33	A*11:01	STLPSGLPK	5.60
	MYO3B	p.K261Nfs*91	A*30:01	KTRSIKILL	5.80
	ELMSAN1	p.I659Sfs*45	DRB1*07:01	TSMPSYQPAPSLPLL	33.44
	ITPRIPL2	p.G141Vfs*138	DRB1*07:01	SRRLWRWSHGAWARS	49.16

Patient 032

Genes	Mutations	HLA	Neoepitopes	Predicted Scores
FAM155B	p.K372T	A*11:01	KTFKESEAPK	26.40
MCF2L	p.S1086Vfs*160	A*11:01	RTVVELLPEK	26.50
NLRP13	p.W619R	A*11:01	RVMEELLKR	38.60
VPS52	p.P126S	B*40:01	TESRFLEQL	48.10
OBSCN	p.P359L	A*11:01	LSTEAAWFK	49.20
SCHIP1	p.R458I	A*11:01	KSIAKIIRRK	55.50
VSTM2A	p.M240K	A*11:01	QSYRVDRFK	125.70
MCF2L	p.S1086Vfs*160	DRB1*09:01	GWVPASSLSVRLGPV	39.35
MANBA	p.D76H	DRB1*09:01	LNYRWVSLHNWTYSK	196.65
ZNF787	p.H140R	DRB1*11:01	FSWSSNLMQRQRIHT	226.06
ALG13	p.S558C	DRB1*11:01	GMPRNSCRFINRHNMP	260.26

Patient 035

Genes	Mutations	HLA	Neoepitopes	Predicted Scores
SUZ12	p.T160Vfs*27	A*02:01	SLSAHLQLV	13.00
ABCB1	p.E1201D	A*02:01	LLDDATSAL	14.90
ABCB1	p.P1028L	A*02:01	LMLNTLEGNV	27.40
SUZ12	p.T160Vfs*27	A*11:01	KMNKILLPWK	48.60
PPARG	p.D177A	A*11:01	CALNCRIHK	63.00
SUZ12	p.T160Vfs*27	DRB1*04:06	SAHLQLVSSTKMISH	159.01
ABCB1	p.E1201D	DRB1*0406	RQPHILLLDDATSAL	211.22
CSNK1A1	p.A9D	DRB1*09:01	KDEFIVGGKYKLVRK	219.44
TP63	p.A259T	B*40:06	REFNEGQIT	227.70
PPARG	p.D177A	DRB1*04:06	RTIRLKLIYDRCALN	479.82

Patient 036

Genes	Mutations	HLA	Neoepitopes	Predicted Scores
IRGC	p.D352N	DRB1*07:01	VLRLYSQSSNGAMRVA	109.61
PRKG2	p.W296L	DRB1*07:01	IKKHRWLNGFNLEGL	251.48
XAB2	p.G246W	DRB1*07:01	DAIIRWGLTRFTDQL	258.57
MAGEC3	p.L467R	DRB1*12:01	EKVAELVQFRLLKYQ	278.43
CATSPERG	p.N203I	DRB1*12:01	RFQMIINGFLKRDRD	328.86
CYHR1	p.T186A	DRB1*12:01	VGSWAYCRALHAQRL	336.01
ZNF254	p.D607Y	B*15:18	NRSSHLTTY	86.1
ANP32E	p.*228Wext*15	C*06:02	LRPDSLMFL	96.1
MCPH1	p.D203H	B*15:18	SHHNPSNSL	348.3

Patient 039

Genes	Mutations	HLA	Neoepitopes	Predicted Scores
GRHL2	p.N603Tfs*41	A*31:01	RAWWRASRSR	9.10
AIM1L	p.P689L	A*11:01	SLISSLTQK	15.10
RNF165	p.H76R	A*31:01	NTIERFTFPR	15.50
ZNF687	p.E204Afs*14	B*55:02	FPSSFELAQA	21.10
ZNF260	p.Q119R	A*31:01	RMPTIIRHQK	62.10
UCMA	p.R49W	B*35:01	WVDELRREY	121.40
TSPOAP1	p.E102Rfs*9	A*31:01	CQRPRMRKWR	145.40
USP51	p.T73M	B*35:01	EPAPEENLM	174.90
AIM1L	p.P689L	DRB1*04:04	GSEGSLISSLTQKEVVQD	126.15
COG6	p.P34L	DRB1*15:01	ATTCNLLSRKLHKILE	202.08

Patient 041	Genes	Mutations	HLA	Neoepitopes	Predicted Scores
	ARHGEF3	p.C215Y	DRB1:09*01	YDSYYSNQVAAKALL	60.87
	CHD2	p.R621Q	A*11:01	GVDEAHQLK	102.30
	FAT1	p.H397Q	A*11:01	YSQLRYVFK	26.20
	FAT1	p.H397Q	DRB1:09*01	TPVVMVKAIPAYSQL	39.25
	LRP2	P.F2108Lfs*27	C*03:02	LAAQWHLIM	10.50
	LRP2	P.F2108Lfs*27	DRB1:09*01	FIGVILAAQWHLIMR	100.22
	TP53	p.R282W	A*33:03	CACPGRDWR	228.10
	TP53	p.R282W	B*58:01	RVCACPGRDW	152.20

Patient 044	Genes	Mutations	HLA	Neoepitopes	Predicted Scores
	CSMD1	p.L3187R	A*33:03	FFQCKSPFIR	46.20
	RNF43	p.G347Tfs*69	A*33:03	DLVPSCHPR	65.70
	PRKCA	p.G152R	A*33:03	CVINVPSLCR	72.70
	MUC6	p.N1065Kfs*28	B*57:01	RGLRARRMW	82.10
	HELZ2	p.G1037R	A*33:03	AVKEDVVPR	125.10
	ZNF607	p.Q243K	B*57:01	KSIHTGEKPF	135.10
	CLIC1	p.L84Wfs*16	A*33:03	DTNKIEEFWR	170.30
	RIN3	p.K529N	DRB1*13:02	LSNNRKLYKNVVELA	79.42

Patient 045	Genes	Mutations	HLA	Neoepitopes	Predicted Scores
	FAM117A	p.P422L	DRB1*09:01	PRKDLEASKASPLPF	56.66
	SARS2	p.P204A	C*03:04	FQARGHLEI	57.50
	LRP1B	p.D1624Y	DRB1*09:01	YWTYIKTQTIKRAFI	59.86
	HBD	p.R117H	C*03:04	LAHNFGKEF	89.20
	LYZ	p.N45Kfs*27	DRB1*09:01	MDGYRGISLAKLDVF	95.90
	DPP9	p.D496Vfs*5	DRB1*09:01	HLYKVTAVLKSQGYV	101.98
	TP53BP1	p.Y1972Cfs*26	DRB1*15:01	SLKILGLTGFIPCYR	104.36
	LRP1B	p.D1624Y	C*03:04	WTYIKTQTI	111.60
	OR2W1	p.T228M	DRB1*15:01	KAVLRMKSKASQRKA	114.78
	PXDN	p.R1142W	B*40:06	TEWLFSMAHT	173.70
	LYZ	p.N45Kfs*27	A*11:01	TSYKLQCWR	219.80

Patient 049	Genes	Mutations	HLA	Neoepitopes	Predicted Scores
	ABCG8	p.T401M	B*07:02	MPGAVQQFTM	162.60
	AGTPBP1	p.I491L	A*02:01	LLSKEPKPFV	195.30
	DNAH10	p.Q2901H	A*02:01	ILSQIGHEA	204.50
	BOC	p.R571W	C*07:02	GRWPKPEIM	256.70
	ABCG8	p.T401M	DRB1*12:02	FTMLIRRQISNDFRD	316.02
	TACR3	p.R230H	A*24:02	KVMPGHTLCF	319.80
	ARHGEF6	p.F155Y	A*24:02	KYPENQHKV	323.80
	IL17RA	p.R812H	DRB1*15:01	LQRQLLFHQLQKNSG	346.65
	TRIT1	p.D372G	C*07:02	KRSYHLCGL	478.20

Patient 055	Genes	Mutations	HLA	Neoepitopes	Predicted Scores
	ACSM2A	p.G76_K77dup	A*31:01	KGKELMWNFR	14.90
	SPEG	p.P3160L	C*14:02	YIMLSGRSL	20.10
	PHF3	p.S846Ffs*7	A*31:01	RQSVRHFSK	20.80
	AIG1	p.C12W	C*14:02	LWNYKAIEM	56.00

	CYP7B1	p.S126I	A*02:06	KLLEKAFSII	99.40
	BRINP1	p.P99L	A*31:01	MLEFQRSIR	114.80
	FGF6	p.R84W	A*31:01	YLVGIKWQRR	151.80
	CYP7B1	p.S126I	DRB1*15:01	KAFSIIQLQKNHDMN	138.78
	DDX51	p.H216Y	DRB1*15:01	PDLQKQLRAYGISSY	165.92

Patient 056	Genes	Mutations	HLA	Neoepitopes	Predicted Scores
	GIGYF2	p.G523Mfs*33	B*58:01	HTSIGNSVW	8.80
	GIGYF2	p.G523Mfs*33	A*11:01	ATLSLAEIQK	11.50
	ARID1A	p.H2090Tfs*9	A*11:01	ATPPFSRLEK	20.40
	KLF8	p.K67T	C*03:02	ITIEPPEEL	70.20
	STXBP5L	p.G98R	A*11:01	RTGAIRILR	97.80
	GIGYF2	p.G523Mfs*33	DRB1*03:01	KKQKMVRADPSLLGFSV	28.61
	SMARCAD1	p.H895del	DRB1*03:01	HQHRYLRLDGKTQIS	44.46
	EVX2	p.C439F	DRB1*09:01	GSDFGFSAAAPRSES	81.09
	CREB5	p.R97W	DRB1*09:01	EESSKWNISMHNAVGG	134.84
	SCAF1	p.A1119V	DRB1*09:01	ANLVSRAKAQELIQA	181.09
	PIGR	p.V346Rfs*3	A*11:01	SPIQAWQLFR	101.30

Patient 065	Genes	Mutations	HLA	Neoepitopes	Predicted Scores
	TRIO	p.P2445L	C*07:02	ARASLGTLL	194.30
	TRIO	p.P2445L	DRB1*15:01	LGTLLLGKPRAGAAS	324.96
	TRIO	p.P2445L	DRB1*15:01	ASLGTLLLGKPRAGA	462.48
	TAAs				Rank
	Her-2		A*01:01	HLDMLRHLY	0.01
			B*40:01	RELGSGLAL	0.01
			B*51:01	MPNQAQMRI	0.01
			C*14:02	TYLPTNASL	0.01
			C*07:02	FRNPHQALL	0.01
			DRB1*15:01	RQQKIRKYTMRRLLQ	0.08

Patient 066	Genes	Mutations	HLA	Neoepitopes	Predicted Scores
	SBK3	p.E292K	A*30:01	KTRSPPLAV	5.10
	SRCAP	p.Q2769R	A*30:01	RQRGAASTLV	79.20
	AOAH	p.N220K	A*30:01	ESVYPGRRPK	166.70
	C8orf58	p.E18K	A*30:01	KGLARGCIV	206.20
	GLIS1	p.R64Q	A*02:01	SLKQCCVLGL	224.40
	ELMOD2	p.E182K	A*30:01	LINLVYFSK	445.80
	ELMOD2	p.E182K	DRB1*09:01	LVYFSKNYTSEAHQI	253.17
	NRXN1	p.D146N	DRB1*09:01	KSKRRNMTVFSGLFV	343.79
	ULBP2	p.V39I	DRB1*14:05	SLCYDITIIPKFRPG	458.29
	SRCAP	p.Q2769R	DRB1*09:01	RRQRGAASTLVPGVS	471.00

Patient 067	Genes	Mutations	HLA	Neoepitopes	Predicted Scores
	BLID	p.N80Kfs*27	B*57:01	SAMKRKCSW	47.20
	MAP10	p.R301W	B*57:01	AASGCSHRHW	63.60
	CARS	p.D171Gfs*11	A*24:02	KDYFKFGLLL	67.00
	ACOX1	p.M48T	A*11:01	ITWFKNFVHR	109.70
	PTPRJ	p.L1120Efs*20	DRB1*07:01	KKDFIATQGPLPNTE	110.08

TUBB6	p.P243L	DRB1*07:01	SLRFLGQLNADLRKL	167.66
KLHL14	p.D226N	DRB1*07:01	EMRALLNSLPPPVES	171.20

Patient 068	Genes	Mutations	HLA	Neoepitopes	Predicted Scores
	KRT9	p.G16R	A*33:03	SSYLSRSGR	27.30
	OR1N1	p.T93M	C*07:06	TRHHTISYM	55.80
	NPR1	p.E194K	A*11:01	YAYRPGDEK	133.00
	ZNF512B	p.R844W	A*33:03	LAGGKKWGR	136.60
	MED1	p.K395R	A*33:03	SLQGTLVSR	163.10
	PROSER1	p.F408I	DRB1*04:05	STSAPFTSLPISTSS	176.25
	KRT7	p.G43S	A*33:03	SASRPRVAVR	189.00
	KRT7	p.G43S	DRB1*07:01	GLSASRPRVAVRSAY	194.41
	ZNF512B	p.R844W	A*33:03	NLAGGKKWGR	312.90

Patient 108	Genes	Mutations	HLA	Neoepitopes	Predicted Scores
	AK5	p.S477Afs*16	A*30:01	RSRAACLWT	14.50
	ANKRD35	p.R649W	B*39:01	WHREAQQVL	32.70
	SEZ6	p.P340L	B*39:01	LRFQSLPPL	49.10
	PTPRN	p.Q68R	A*30:01	RARPLLQVT	59.20
	PNMAL2	p.E440K	A*30:01	RGLFGGWSK	83.50
	ADAMTS1	p.K922T	A*30:01	STTCGKGYK	165.40
	FUT1	p.D44Y	C*07:02	YRRLVTPPV	179.30
	FUT1	p.D44Y	DRB1*08:02	LCPYRRLVTPPVAIF	53.11
	AK5	p.S477Afs*16	DRB1*15:01	TNRLLQRSRAACLWT	117.18
	PIGS	p.G398R	DRB1*08:02	RLLFRIAQPQLPPKC	161.18
	SEZ6	p.P340L	DRB1*15:01	HQAALRFQSLPPLAG	189.53
	POM121L2	p.M411I	DRB1*15:01	RKIQKSLGPLASPQS	198.20

Patient 123	Genes	Mutations	HLA	Neoepitopes	Predicted Scores
	AJAP1	p.G22R	A*33:03	SIRWPGRPLR	64.90
	ING1	p.Y195Cfs*13	A*33:03	EPTCTVCATR	68.20
	BAZ2A	p.R1118H	A*33:03	VSLGQDHYR	170.70
	SEMA5B	p.T251M	A*33:03	AAMVIDFSGR	180.90
	OR14J1	p.R51H	C*06:02	HRLHSPMYY	257.50
	SALL1	p.R1213C	B*13:02	TNFCFTRFV	390.10
	KIAA1468	p.A96V	A*33:03	IDVIAAQLLR	403.80
	OR51T1	p.L188R	DRB1*07:01	IRPAVFLLPRLVAIN	179.61
	FLNA	p.P2328L	DRB1*07:01	SPFVVPVASLSGDAR	241.71
	EZH2	p.A692V	DRB1*07:01	IRFVNHSVNPNCYAK	291.29
	AJAP1	p.G22R	DRB1*07:01	LRSHAWILIAMFQLA	309.57

Patient 124	Genes	Mutations	HLA	Neoepitopes	Predicted Scores
	GABRA1	p.P124L	A*02:01	NLMASKIWTL	12.70
	XYLB	p.E514Mfs*31	A*02:01	SLLPLPAQI	40.90
	CPNE4	p.G157V	C*03:03	TAVKSSITV	46.20
	RYR2	p.L885Q	B*48:01	KQAENIHEL	74.00
	PDE7B	p.F75Sfs*24	A*02:01	LLSSKILPCI	99.10
	CPNE4	p.G157V	A*02:01	SLLKHGNTAV	119.30
	SH2D4A	p.V4L	A*02:01	RLIEPPCPGA	180.20

KAZN	p.E76K	DRB1*11:01	MQQLYATLESRKEQL	176.26

Patient 125

Genes	Mutations	HLA	Neoepitopes	Predicted Scores
BCORL1	p.S514L	C*12:02	FSVARPLTL	15.10
CTNND2	p.S242L	A*02:01	GLSPIRVTL	69.10
EFHC2	p.R280W	A*02:01	KMFLWRSKL	123.40
FBXW7	p.G644R	A*68:01	NFVITSSDDR	144.80
KCMF1	p.F78Tfs*22	A*02:01	YLSLLWKNGL	182.60
WNK3	p.T489M	A*68:01	DRVMPIKKTR	248.80
AHNAK2	p.H3225Y	DRB1*15:01	AGLKGYLPKLQMPSF	302.56
BCORL1	p.S514L	DRB1*15:01	AFSVARPLTLDSKLV	311.30

Patient 126

Genes	Mutations	HLA	Neoepitopes	Predicted Scores
MKRN3	p.S360Y	B*15:07	RQFENRIVKY	27.20
PIGR	p.V367Gfs*9	C*03:03	VAGGSGRAL	78.60
MYH9	p.R639W	A*02:01	KTWKGMFRTV	82.20
YAE1D1	p.F167L	A*02:01	RLCENNAEL	88.00
PRPH	p.S75L	A*02:01	ALLRLPLERL	160.20
TRIM32	p.C120Y	A*02:01	VLYEPCREA	178.00
IGF2BP1	p.R209W	A*02:01	EIMKKVWEA	179.90
DUSP13	p.T120M	DRB1*13:12	ADFIHRALNMPGAKV	309.40
ZNF331	p.S340L	DRB1*13:12	FRWGLSLVKHERIHT	322.77
OR12D2	p.P286L	DRB1*04:03	VLNLLIYTLRNKEVK	338.26

Patient 139

Genes	Mutations	HLA	Neoepitopes	Predicted Scores
PSG8	p.P281L	A*03:01	SLLVSPRVK	108.90
SPATA5L1	p.P52L	A*02:01	SLLDGGSCL	110.00
SHISA7	p.G345S	C*03:04	RSTLPLHAL	163.50
CDH1	p.D367Pfs*25	DRB1*13:02	TIILRSSIPPRTRVR	30.13
PDE11A	p.P157Lfs*23	DRB1*07:01	KAEVDKFKAANILWC	79.92
CCNB1IP1	p.P243L	DRB1*07:01	QFRPFFAGSLTAPEP	92.90
PSG8	p.P281L	DRB1*07:01	WLNGQSLLVSPRVKR	200.67
SLC24A4	p.G5R	DRB1*13:02	LRRTLRPLKVRRRRE	275.97

Patient 155

Genes	Mutations	HLA	Neoepitopes	Predicted Scores
KLHL7	p.T246M	A*32:01	KMVQAEPLI	115.80
GALR1	p.K242M	B*15:01	MSKKSEASM	200.40
BDP1	p.T1285A	A*31:01	AGKENFRER	257.70
KLHL7	p.T246M	DRB1*15:01	ISKNFLSKMVQAEPL	271.47
NBEA	p.R306H	B*35:01	HALNVFHYL	322.80
TAAs				
GPC-3			RELIQKLKSFISFYS	
Survivin			RAIEQLAAM	
Survivin			HRISTFKNWPFLEGC	
MUC1			STAPPAHGVTSAPDTRPAPGSTAPP	
MUC1			SVSDVPFPF	
