# Supplementary material for: Benefits of an Immunogenic Personalized Neoantigen Nanovaccine in Patients with High‐Risk Gastric/Gastroesophageal Junction Cancer
Source: Adv Sci (Weinh). 2022 Nov 9;10(1):2203298. doi: 10.1002/advs.202203298 (PMC9811442; doi:10.1002/advs.202203298)
Supplement: Supplementary file 4 — Supplemental Table 3 [file ADVS-10-2203298-s001.pdf]

Nanovaccine-related adverse events (AEs)				
	Grade 1	Grade 2	Grade 3	Grade 4
Local skin reactions	12	2	/	/
Fever	4	1	/	/
Myalgia	1	2	/	/
